# Additional risk of diabetes exceeds the increased risk of cancer caused by radiation exposure after the Fukushima disaster

**DOI:** 10.1371/journal.pone.0185259

**Published:** 2017-09-28

**Authors:** Michio Murakami, Masaharu Tsubokura, Kyoko Ono, Shuhei Nomura, Tomoyoshi Oikawa

**Affiliations:** 1 Department of Health Risk Communication, Fukushima Medical University School of Medicine, 1 Hikarigaoka, Fukushima, Fukushima, Japan; 2 Radiation Medical Science Center for the Fukushima Health Management Survey, Fukushima Medical University, 1 Hikarigaoka, Fukushima, Fukushima, Japan; 3 Department of Radiation Protection, Minamisoma Municipal General Hospital, 2-54-6 Takami, Haramachi, Minamisoma, Fukushima, Japan; 4 Department of Radiation Protection, Soma Central Hospital, 3-5-18 Okinouchi, Soma, Fukushima, Japan; 5 Research Institute of Science for Safety and Sustainability, National Institute of Advanced Industrial Science and Technology (AIST), 16–1, Onogawa, Tsukuba, Ibaraki, Japan; 6 Department of Epidemiology and Biostatistics, School of Public Health, Imperial College London, Norfolk Place, London, United Kingdom; 7 Department of Global Health Policy, Graduate School of Medicine, The University of Tokyo, 7-3-1, Hongo, Bunkyo, Tokyo, Japan; Northwestern University Feinberg School of Medicine, UNITED STATES

## Abstract

The 2011 Fukushima disaster led to increases in multiple risks (e.g., lifestyle diseases and radiation exposure) and fear among the public. Here, we assessed the additional risks of cancer caused by radiation and diabetes related to the disaster and the cost-effectiveness of countermeasures against these conditions. Our study included residents of the cities of Minamisoma and Soma (10–40 km and 35–50 km north of the Fukushima Daiichi (N° 1) Nuclear Power Station, respectively). We used the loss of life expectancy (LLE) as an indicator to compare risks between radiation exposure and diabetes. We also estimated the cost-effectiveness of radiation-related countermeasures, including restricted food distribution, decontamination, and whole-body counter tests and interventions. Metformin therapy was selected as a representative management for diabetes. The diabetes-related LLEs among residents were 4.1 (95% confidence interval: 1.4–6.8) ×10^−2^ years for the whole population and 8.0 (2.7–13.2) ×10^−2^ years for 40s to 70s in a scenario that considered the additional incidence of diabetes during the first 10 years. The cancer-related LLEs caused by lifetime exposure to radiation were 0.69 (2.5–97.5 percentile: 0.61–0.79) ×10^−2^ years for the whole population and 0.24 (0.20–0.29) ×10^−2^ years for 40s to 70s. The diabetes-related LLEs among residents in the above-mentioned scenario were 5.9-fold and 33-fold higher than those attributed to average radiation among the whole population and among the 40s to 70s age groups, respectively. The costs per life-years saved of the radiation countermeasures (i.e., restricted food distribution, decontamination, and whole-body counter tests and interventions) were >1 to >4 orders of magnitude higher than those of general heath checkups and conventional management for diabetes. Our findings indicate that countermeasures to mitigate diabetes are warranted. Policy-makers’ and individuals’ understanding of multiple risks after any disaster will be essential to saving the lives of victims.

## Introduction

The concepts of risk and risk comparison were first introduced in the 1660s through two publications that offered insights into unreasonable fears: *Natural and Political Observations Made upon the Bills of Mortality* by John Graunt and *Port-Royal Logic* by Arnauld and Nicole [1,2]. Subsequently, individuals and societies have used risk comparisons and risk trade-off assessments to support decision-making [3].

After a disaster, risk trade-offs, whereby the avoidance of one risk results in a much greater increase of another risk, often emerge as a consequence of fear [4]. The Tokyo Electric Power Company (TEPCO) Fukushima Daiichi (N° 1) Nuclear Power Station (NPS) accident that followed the Great East Japan Earthquake on March 11, 2011 led to multiple social risks and trade-offs. The Japanese government ordered a full evacuation of residents living within 20 km of the Fukushima Daiichi NPS and within 10 km of the Fukushima Daini (N° 2) NPS on March 12, 2011, and ordered the further evacuation of residents who were likely to have received an additional effective dose (attributable to Fukushima emissions) of ≥20 mSv/year on April 22. Although the radiation exposure is reported to have been limited [5], fear among the residents has not been dispelled [6,7]. In addition, the evacuations forced changes in evacuees’ lives, including losses of homes and employment, separation of families, and disruptions of health services. Although post-disaster studies have revealed significant increases in the prevalence of lifestyle diseases, especially diabetes, among evacuees [8–11], the prevalence of diabetes in Japan did not change significantly from before to after the disaster [12]. Diabetes is a significant risk factor for various diseases, including cancer and all-cause mortality [13], and has spread worldwide [14]. Although citizens perceive radiation risk to be the most dreadful of various hazards [15], anxieties regarding lifestyle diseases, including diabetes, did not increase in Japan after the disaster [16]. However, the persistent silent risk of diabetes could potentially pose a serious health risk. Accordingly, policy-makers must address both real public health dangers and fears [17].

Radiation-related countermeasures have been applied to reduce not only risk, but also anxiety; as a result, excessive fears have led to demands for expensive radiation-related countermeasures (e.g., decontamination at a cost of 2.53–5.12 × 10^12^ Japanese yen (JPY)) [18]. As financial resources are limited, these high expenditures could limit other effective countermeasures and eventually result in losses of life. A comparison of various potential risks and the cost-effectiveness of countermeasures against those risks would therefore help to protect the public from real dangers. In addition, the lack of a multiple-risk comparison could result in the overestimation of a specific risk among the public (also known as “out of sight, out of mind” [19]); furtheremore, the provision of objective numerical data and risk comparison information is the most important risk communication method for reducing fear and improving comprehension [20–22] and can thus be used to mitigate fear among the public. The International Commission on Radiological Protection (ICRP) uses the concepts of “optimization of protection” and “as low as reasonably achievable” [23], and quantitative evaluations of multiple risks and the cost-effectiveness of various countermeasures are important when determining the applications of these concepts. In the future, decision-making and risk communication processes that are supported by a better understanding of risk comparison and cost-effectiveness will save lives in Fukushima and improve emergency preparedness. Despite these advantages, a multiple risk comparison that includes cancer mortality caused by radiation and medical risks has not been performed because of the lack of interdisciplinary scientific research.

We therefore assessed the cancer risks related to radiation and diabetes after the Fukushima disaster and compared these with additional risks attributable to the disaster. We then estimated the cost-effectiveness of the radiation countermeasures implemented after the disaster in comparison with those of general health checkups and subsequent conventional management for diabetes. In this study, we studied residents in the cities of Minamisoma and Soma, located 10–40 km and 35–50 km north of the Fukushima Daiichi NPS, respectively (S1 Fig). Part of Minamisoma City was placed under an evacuation order, whereas Soma City was not.

## Methods

### Ethics approval

Ethical approval for this study was granted by the ethics committee of the Minamisoma Municipal General Hospital (authorization number 28–02).

### Basic concept

Of the 108 473 total individuals residing in the cities of Minamisoma and Soma on March 1, 2011 [24], approximately 17 000 were mandatorily evacuated. We previously reported the risks of internal and external radiation exposure, nursing-home evacuation, and lifestyle diseases in this population [10,25–28]. To compare the additional risks of cancer caused by radiation exposure and diabetes (i.e., post-disaster increases), we used the loss of life expectancy (LLE) as a risk indicator in accordance with our previous study [29]. The LLE provides two major advantages over other risk indicators such as mortality rates or quality-adjusted life years: (1) LLEs can be quantitatively estimated from objective measurable data, and (2) differences in the timing of radiation exposure- and diabetes-related mortality events can be expressed in consideration of the age reached at the time of death.

The overall diabetes-related risk depends on the future incidence of each age and sex group; however, these rates are unknown. Accordingly, to conduct a prior risk assessment that would demonstrate the importance of anti-diabetes measures, we set three scenarios for diabetes and assessed the additional risks of cancer caused by radiation and diabetes during three time stages: years 1–4 after the disaster (March 11, 2011–March 10, 2015); years 5–10 (March 11, 2015–March 10, 2021); and year 11 and beyond (beginning March 11, 2021). For radiation-related cancer, the risks during these three time intervals were estimated and the sum of the risk due to lifetime exposure was estimated. The 2.5–97.5 radiation risk percentiles were also estimated based on dose distribution (see details in “Variations in LLE”). For diabetes, the risk during years 1–4 was considered in Scenario 1, whereas the risks in two time intervals (years 1–4 and years 5–10) were estimated in Scenarios 2 and 3 (see details in “Diabetes risk”). These scenarios were set as benchmarks for a prior risk assessment. This analysis did not aim to predict the actual probability of mortality, but rather to provide useful information for social and individual decision-making. This study demonstrated the degree of diabetes risk by calculating this to avoid overestimation, and calculated radiation risk to avoid underestimation.

### Radiation risk

#### Estimation of additional doses

To estimate the doses to residents of Minamisoma and Soma, we considered three pathways of exposure to artificial radionuclides released from the Fukushima Daiichi NPS—external exposure, inhalation and ingestion—in accordance with previous studies [5,30,31]. The details are described in S1 Method.

#### LLE due to cancer caused by radiation

The lifetime attributable risks (LARs) of mortality and the LLEs corresponding to cancer caused by radiation were calculated in accordance with our previous study [29]. The details are described in S1 Method.

#### Variations in LLE

Variations in the LARs of mortality and LLEs were estimated using a Monte Carlo simulation performed with Crystal Ball software (Oracle, Redwood City, CA, USA). Here, we considered variations in the organ doses. All doses were assumed to follow a log-normal distribution. The relative standard deviation was set at 40%, based on data measured in Minamisoma [28]. Variations in cumulative organ doses were calculated for years 1–4, years 5–10, and years 11 and onwards and for the total period, and cumulative organ doses were distributed according to annual organ doses; in other words, residents who received high doses were recorded as having a high dose every year. Variations in the LARs of mortality and LLEs were estimated from the input organ dose data for the Monte Carlo simulation. This simulation was performed 100 000 times for each age and sex at each time stage.

#### Validation of doses

We validated the assessment by comparing the estimated values from external exposure with the monitored data of infants, children, and pregnant women in Minamisoma. The estimated values for children aged 10 years at the time of the disaster were 0.33 mSv in June–August 2012, 0.31 mSv in September–November 2012, and 0.29 mSv in December 2012–February 2013, or approximately double the observed values (median: 0.17, 0.17 and 0.15 mSv, respectively) [32]. For children in this age group, the 2.5 and 97.5 percentile effective doses during year 2 were 0.74 and 3.36 mSv/year, respectively, or were 1.8–3.7 fold of the measured data (0.2–0.3 and 1.8–1.9 mSv/year) [32]. As the doses to residents were slightly higher in Minamisoma than in Soma, the estimated values were considered to be higher than the actual values (see “Sources of uncertainty”).

### Diabetes risk

#### Participants

Previous reports have described increases in the diabetes risk after a disaster, especially among evacuees [8–10]. To assess the diabetes risk, we used data from public health checkups administered by the cities of Minamisoma and Soma between 2008 and 2014 [10]. These public health checkups were available only for those aged 40–74 years. We included participants who attended more than one checkup both before and after the disaster to exclude those who moved to or from these cities after the disaster. We defined diabetes as a glycated hemoglobin (HbA1c) level of ≥6.5% or the use of antihyperglycemic agents. For individuals who participated in multiple checkups during 1 year, we used data from the first checkup during that year. Our previous study reported significant increases in the prevalence of diabetes in both Minamisoma and Soma after the disaster [10], whereas a corresponding significant change in the nationwide prevalence of diabetes was not observed in Japan [12]. The prevalence of diabetes before and after the disaster is summarized in S1 and S2 Tables. The demographic characteristics of the 2011 participants, including unobserved factors, may have differed from those in other post-disaster years (e.g., people who experienced significant disruptions in their lives just after the disaster might have been less able to participate in checkups in 2011), which would limit the generalizability of the 40–74 years age cohort in Minamisoma; accordingly, we excluded the 2011 data to avoid potential participant selection bias. Overall, 14 869 (5995 men, 8874 women) and 12 981 (5197 men, 7784 women) participants were included before (2008–2010) and after the disaster, respectively. Of these, 15.1% lived in mandatory evacuation areas at the time of the disaster.

#### Scenarios

**Fig 1:**
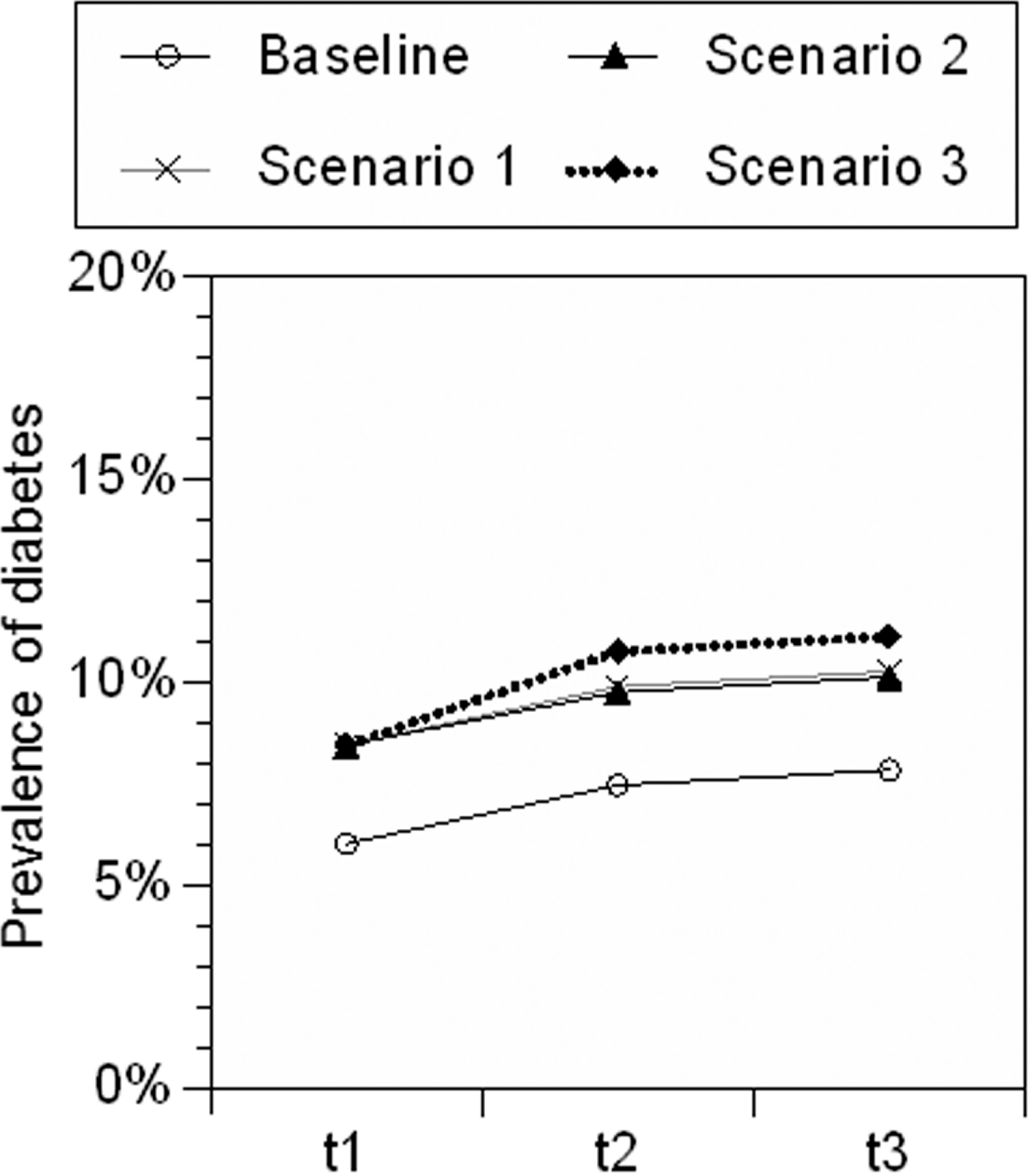
Prevalence of diabetes at baseline and in each of three scenarios in the whole population. t1: years 1–4; t2: years 5–10; t3: years 11–14.

The diabetes risk was estimated for the 40s to 70s age groups at the time of the disaster (total: 55 798 individuals) using health check data collected in these cities from 2008 to 2014 and from Japanese cohort data regarding all-cause mortality [13] (see details in “LLE due to diabetes”). As described in “Basic concept”, the risk of diabetes depends on the future incidence, which is currently unknown. LLEs were calculated under three scenarios used as benchmarks: in Scenario 1, additional diabetes emerged only in the first 4 years (March 11, 2011–March 10, 2015) and reflected the “emergent incidence” of diabetes that would not occur if there were no disaster; in Scenario 2, an increasing diabetes prevalence continued for 6 subsequent years (March 11, 2015–March 10, 2021); and in Scenario 3, the diabetes prevalence reflected the worst case outcome from Scenarios 1 and 2, and only the additional incidence of diabetes (i.e., emergent incidence) was considered during the first 10 years. An increase in the prevalence of diabetes could be attributed not only to emergent incidence, but also to premature incidence (i.e., diabetes that would have occurred later in the absence of a disaster). In Scenario 2, the additional incidence of diabetes during years 5–10 among men aged 40s and 50s at the time of the disaster yielded negative values (S3 Table) because the increased prevalence of diabetes among men after the disaster was higher among those aged 40–43 and 50–53 years than among those aged 44–49 and 54–59 years, respectively (S1 Table). One possible interpretation of this result is that the increase of diabetes among men in 40s and 50s during years 1–4 was attributable to premature incidence rather than emergent incidence. The prevalence rates of diabetes in the whole population during the stated time intervals, at baseline (i.e., before the disaster), and in each of the three scenarios are shown in Fig 1. Details for each age and sex group are shown in S1–S4 Tables. As public health checkup data from Minamisoma and Soma were available for the first 4 years, we divided the scenarios into bands of 4 and 6 years.

#### LLE due to diabetes

We estimated the LARs of mortality and LLEs due to diabetes using the additional incidence of diabetes after the disaster, the hazard ratio (HR) of diabetes for all-cause mortality from a Japanese cohort study [13], and the Japanese survival probability [33]. The details are described in S1 Method.

### Cost and effectiveness of countermeasures

We approximated the cost-effectiveness of three radiation-related countermeasures—restricted food distribution, decontamination, and whole-body counter tests and interventions—and of health checkups and metformin therapy for diabetes. Here, we targeted the cost-effectiveness of radiation-related countermeasures during the early stage, when they would have the greatest effect. Metformin therapy was selected as a representative management for diabetes because it is practically applied and was reported to significantly reduce mortality in a randomized controlled trial of patients with high body mass index (BMI) values [34]. The effects of countermeasures were estimated by using the life-years saved (LYS; i.e., reduction of LLE) as an indicator. The details are described in S1 Method.

## Results

### Risks of diabetes and radiation-induced cancer

The LLEs and LARs of cancer caused by radiation exposure and diabetes for the whole population and for the 40s to 70s age groups were compared (Table 1, S5–S9 Tables). Radiation exposure occurred mainly during the first year and rapidly decreased thereafter. The lifetime radiation exposure effective doses up to 89 years for individuals who were 0 years old at the time of the disaster, the whole population, and the 40s to 70s age groups were 23, 16, and 15 mSv, respectively (S2 Fig). The LLEs due to radiation exposure in the whole population were 0.37 × 10^−2^ years for years 1–4, 0.14 × 10^−2^ years for years 5–10, 0.18 × 10^−2^ years for year 11 and beyond, and 0.69 × 10^−2^ years throughout a lifetime. Because the LLEs due to radiation exposure decreased with increasing age at the time of the disaster (S5 Table), the total lifetime LLEs were lower for the 40s to 70s age groups (0.24 × 10^−2^ years), compared to those for the whole population. The 2.5–97.5 percentile ranges of total lifetime LLEs due to radiation exposure were 0.61–0.79 for the whole population and 0.20–0.29 for the 40s to 70s age groups, respectively. In contrast, among the 40s to 70s age groups, the LLEs due to diabetes throughout their lifetimes were 5.0 (95% confidence interval: 0.5–9.5) × 10^−2^ years in Scenario 1, 5.5 (−0.9–12.0) × 10^−2^ years in Scenario 2, and 8.0 (2.7–13.2) × 10^−2^ years in Scenario 3. The diabetes risk during year 11 and beyond was not determined because of the high level of uncertainty in the future; however, among the 40s to 70s age groups, the average total LLEs due to diabetes for Scenarios 1, 2, and 3 were 21, 23, and 33 times higher, respectively, than those attributed to average lifetime radiation exposure. These differences highlight the much higher risk of diabetes. Because the diabetes risk was calculated only for the 40s to 70s age groups at the time of the disaster, the total LLEs due to diabetes in the whole population were diluted to 2.6 (0.3–4.9) × 10^−2^ years in Scenario 1, 2.9 (−0.5–6.2)× 10^−2^ years in Scenario 2, and 4.1 (1.4–6.8) × 10^−2^ years in Scenario 3; notably, these average values were 3.7, 4.1, and 5.9 times higher, respectively, than those attributed to the average lifetime radiation exposure. Although the future prevalence of diabetes is unclear, the average risks of diabetes for the 40s to 70s age groups and the whole population for year 5 and beyond exceed those of radiation by 31 times and 4.8 times in Scenarios 2 and 3, respectively. In summary, the diabetes risk was not only considerable during the first 4 years after the disaster, but will also potentially be strikingly high in the future. Countermeasures to mitigate diabetes are therefore warranted.

**Table 1:** Losses of life expectancy (LLEs) due to radiation-related cancer or diabetes in the whole population or among the 40s to 70s age groups (10^−2^ years). The additional post-disaster risks of both radiation exposure and diabetes were assessed. The values in parentheses for radiation exposure represent the 2.5–97.5 percentiles based on dose distributions. The values in parenthesis for diabetes represent 95% confidence interval.

	Whole population				40s–70s			
	Radiation exposure	Diabetes			Radiation exposure	Diabetes		
		Scenario 1	Scenario 2	Scenario 3		Scenario 1	Scenario 2	Scenario 3
Years 1–4	0.37 (0.31–0.46)	2.6 (0.3–4.9)	1.3 (−1.7–4.3)	2.6 (0.3–4.9)	0.15 (0.11–0.19)	5.0 (0.5–9.5)	2.6 (−3.2–8.4)	5.0 (0.5–9.5)
Years 5–10	0.14 (0.11–0.17)	–	1.5 (0.1–2.9)	1.5 (0.1–2.9)	0.052 (0.040–0.068)	–	3.0 (0.2–5.7)	3.0 (0.2–5.7)
Years 11–	0.18 (0.14–0.22)	–	–	–	0.044 (0.033–0.058)	–	–	–
Total	0.69 (0.61–0.79)	2.6 (0.3–4.9)	2.9 (−0.5–6.2)	4.1 (1.4–6.8)	0.24 (0.20–0.29)	5.0 (0.5–9.5)	5.5 (−0.9–12.0)	8.0 (2.7–13.2)

### Costs and effectiveness of countermeasures

The costs and effectiveness of radiation countermeasures and general health checkups and conventional management for diabetes were calculated for the whole population (Table 2, S10–S14 Tables). Of the countermeasures, decontamination yielded the largest increase in the life-years saved (LYS) (4.8 × 10^−3^ years), followed by restricted food distribution (1.3 × 10^−5^ years), and whole-body counter tests and interventions (6.1 × 10^−8^ years). The effectiveness of whole-body counter tests and interventions was estimated from a group of 8 individuals (from among 30,622 screened participants) who were exposed to ^134^Cs and ^137^Cs doses exceeding 50 Bq/kg and subsequently received interventions, and the costs were calculated based on all screenings. We did not include the effectiveness for other participants who were exposed to lower doses of ^134^Cs and ^137^Cs and did not receive interventions but did voluntarily reduce radiocesium intake through changes in dietary habits, or the effectiveness of risk communication, including reduced anxiety [35]. Although general health checkups and conventional management for diabetes are known to effectively reduce risk only among patients with diabetes and a high BMI, these measures increased life-years by >0.045 years among the whole population. General health checkups and conventional management for diabetes were associated with a cost per life-years saved (CPLYS) of <7.4 million JPY/year, which may be comparable to or even better than the values associated with disease prevention (median: 3.3 million JPY/year) and medical treatment (0.97 million JPY/year) [36]. The CPLYS for restricted food distribution, decontamination, and whole-body counter tests and interventions were approximately >1, >1–2 and >4 orders of magnitude higher, respectively, than the CPLYS for general health checkups and conventional diabetes management. Overall, health checkups and conventional management for diabetes yielded the highest level of effectiveness and lowest CPLYS relative to radiation-related countermeasures. The costs of restricted food distribution and decontamination were borne by the central government or charged to the TEPCO and were eventually passed to consumers in Tokyo and the surrounding areas, whereas the costs of whole-body counter tests and interventions and of general health checkups and conventional management for diabetes were apportioned mainly to local municipalities and partly to residents (S10 Table). Because the financial budget for additional risks related to the Fukushima Daiichi NPS accident is limited to radiation-related countermeasures and cannot be used for general health promotions (including diabetes countermeasures), policy-making has not emphasized cost-effectiveness. Overall, this has resulted in a lack of incentive to promote cost-effective countermeasures.

**Table 2:** Costs and effectiveness of early countermeasures against radiation exposure and diabetes (metformin therapy). LYS: life-years saved; CPLYS: cost per life-years saved. The effectiveness of whole-body counter tests and interventions was estimated from a total of 8 individuals who exceeded 50 Bq/kg of ^134^Cs and ^137^Cs and received interventions among 30,622 screened participants, and the costs were calculated based on all the screenings.

	Restriction of food distribution	Decontamination	Whole-body counter tests and interventions	General health checkups and conventional management for diabetes
LYS (years)	1.3 × 10^−5^	4.8 × 10^−3^	6.1 × 10^−8^	>4.5 × 10^−2^
Per-capita cost (JPY)	7.5 × 10^2^	1.1 × 10^6^	7.4 × 10^3^	<3.4 × 10^5^
CPLYS (JPY/year)	5.6 × 10^7^	2.4 × 10^8^	1.2 × 10^11^	<7.4 × 10^6^

## Discussion

The results of this study have implications both for future policy-making in Fukushima and for general emergency preparedness. First, the additional risk of diabetes was higher than that of radiation exposure-induced cancer, especially in Scenario 3. The future prevalence of diabetes in Fukushima is not clear; however, from March 2015 onwards, the average additional risk of diabetes among the 40s to 70s age groups exceeded that of radiation by 31-fold, depending on the scenario. Although the causes of emergent diabetes among evacuees remain unclear, improvements in dietary and exercise habits are keys to preventing diabetes in general [37]; more habitual physical activity was found to contribute to a large reduction in future all-cause mortality among patients with diabetes [38]. For both policy-makers and individuals, a better understanding of the multiple potential risks after the disaster will reduce fears biased towards radiation and help to protect the public from greater dangers. Note that the future prevalence of diabetes and its correspondent risks could be higher or lower than those obtained from the scenarios used in this study. This study will prove successful if social advances in the comprehensive understanding of multiple risks after the disaster lead to a lower risk of diabetes relative to the values calculated herein, with a consequent increase in lives saved.

This study has additional implications with regard to multiple risks after a disaster. The lifetime radiation exposure doses in Minamisoma and Soma were 16 mSv for the whole population and 15 mSv for the 40s to 70s age groups, and the level of averted radiation exposure in these cities as a result of evacuation was negligibly small or even negative [5]. Given the average ratio of the LLEs of diabetes to that of radiation-induced cancer (in the range from 3.7 to 5.9 times for the whole population and from 21 to 33 times among the 40s to 70s age groups, depending on scenario), the proportion of evacuated residents (15.1%) and the more serious diabetes risk incurred by evacuees relative to non-evacuees (hazard ratio: 1.399 [9]; odds ratio: 1.14 [10]), the LLE due to diabetes risk among non-evacuees in the whole population would be equivalent to a radiation exposure of 60–90 mSv. Among the 40 to 70s age group, the risk would be equivalent to a radiation exposure of 300–500 mSv. The LLE due to the added risk of diabetes directly caused by evacuation would be equivalent to a radiation exposure of 10–40 mSv among the whole population and of 40–200 mSv among the 40s to 70s age groups. According to our results, the essential problem after a nuclear power plant disaster is the potential for an associated societal change to trigger greater dangers; in other words, mandatory evacuation may cause greater health risks than the avoidance of radiation at a lifetime exposure of several tens of mSv. We do not mean to suggest the decision to evacuate was inappropriate. The lack of knowledge of the post-evacuation diabetes risk and the fears of radiation are natural and should not be blamed. In hindsight, however, our risk analysis shows that additional evacuation-related risks should be fully considered in the context of emergency preparedness. Furthermore, more cost effective general health countermeasures, rather than radiation-related countermeasures, should be sought. A careful consideration of expense defrayers and a flexible budget that allows cost-effective comprehensive countermeasures are needed to save the lives of disaster victims. Regarding societal decision-making, the greatest lesson learned from Fukushima is that improved understanding and preparedness in terms of health deterioration (which is worsened by evacuation) and radiation are crucial to protecting the public from greater dangers.

### Sources of uncertainty

Various factors led to the overestimation of the radiation-induced cancer risk. First, external exposure doses were based on values from contaminated areas in Minamisoma. Similarly, inhalation doses were based on values from Minamisoma. Therefore, the radiation risk among the whole population in Minamisoma and Soma was likely overestimated, as described in “Validation of doses” where the estimated values approximately doubled the observed values. In Minamisoma, the median values of ^137^Cs in the soils and the ambient dose equivalent were 1.6 and 1.9 times higher than those in Soma [39]. As the population of Minamisoma was 1.88 times greater that of Soma, this might have led to a dose overestimation by a factor of ~2.5. Second, environmental decay (except physical decay), including weathering effects, was not considered. Third, the use of linear–quadratic dose–response models at low doses can lead to overestimations of actual mortality rates. The assessment of radiation risks at low doses, first demonstrated in ICRP Publication 1 as a *de facto* linear non-threshold theory [40], is a tool for protection and risk management rather than for predicting the actual probability of cancer mortality [2]. Fourth, the estimated cancer mortality did not reflect possible future advances in medical cancer care. The LARs of mortality of associated with all solid cancers and with leukemia for 20–60 years consequent to 20 mSv radiation exposure corresponded to 55% to 73% of the incidence rate [29]. Therefore, the estimated LLEs could decrease with medical advances.

Various factors led to the underestimation of diabetes risks. First, the HRs of diabetes used in this study did not include participants with cardiovascular disease, chronic liver disease, kidney disease, or any type of cancer at baseline. Because these participants are thought to have a higher prevalence of diabetes, the HRs of diabetes for the whole population, including participants with the above diseases, would be higher than the values used in this study. Therefore, we consider our results to be underestimation. Second, the HRs of 1.59 and 2.00 in a previous study [13] covered the whole follow-up period, including the first 15 years after the incidents, and therefore the use of these values for >15 years after the incident also underestimated the results. Third, any increase in the prevalence of diabetes from March 2021 onwards was not considered. If this increase continues beyond March 2021, the diabetes risk would be higher than the value reported in this paper (i.e., the values provided from this study are potentially underestimated). However, as with the radiation risk, we did not consider possible advances in medical care. Fourth, there was uncertainty about the extent of incidence of diabetes, especially for the 40 to 50s age group, because these population data used in this study were limited as a few hundred (S1 and S2 Tables). Fifth, although we assessed the risk due to diabetes, increases in other health risks, including hyperlipidemia [10], hypertension [41], and psychological distress [42] were observed among evacuees after the disaster. These risks were not evaluated in this study and a further study is required.

The cost-effectiveness of health checkups and metformin therapy for diabetes remained somewhat uncertain. We used data regarding the effects of metformin therapy in the UK, but note that those may not be fully applicable to the Japanese setting. In this regard, however, we conservatively calculated the effect as the difference between metformin therapy and other types of conventional therapy, rather than between therapy and no therapy, and accordingly the effect was likely higher than estimated. We also applied the cost of metformin therapy for diabetes in the UK, which was approximately one-third of the costs of multiple insulin injection therapy (4.18 million JPY/patient) or conventional insulin injection therapy in Japan (4.40 million JPY/patient) [43]. However, these values would not greatly influence the results because most of the costs were apportioned to health checkups (S10 Table). As the cost-effectiveness differed by orders of magnitude among the evaluated countermeasures, we do not believe the uncertainty to have influenced the implications revealed in this study.

## Supporting information

S1 Method (PDF)

S1 Table Numbers of male patients with diabetes and total participants, and the prevalence before and after the disaster. The values in parenthesis represent 95% confidence interval.
(PDF)

S2 Table Numbers of female patients with diabetes and total participants, and the prevalence before and after the disaster. The values in parenthesis represent 95% confidence interval.
(PDF)

S3 Table Prevalence and additional incidence of diabetes at baseline and in each scenario among men. Scenario 1: Emerging diabetes occurred only during the first 4 years. Scenario 2: Emerging diabetes occurred during the first 10 years. Premature incidence was considered. Scenario 3: Diabetes prevalence combined the worst cases of scenarios 1 and 2 (only emerging diabetes was considered, and no premature incidence was assumed). The values in parenthesis represent 95% confidence interval.
(PDF)

S4 Table Prevalence and additional incidence of diabetes at baseline and in each scenario among women. Scenario 1: Emerging diabetes occurred only during the first 4 years. Scenario 2: Emerging diabetes occurred during the first 10 years. Premature incidence was considered. Scenario 3: Diabetes prevalence combined the worst cases of scenarios 1 and 2 (only emerging diabetes was considered, and no premature incidence was assumed). The values in parenthesis represent 95% confidence interval.
(PDF)

S5 Table LLEs among age groups as a result of radiation exposure-induced cancer. The additional post-disaster risk was assessed. M: men; W: women. Values in parentheses represent the 2.5–97.5 percentile range.
(PDF)

S6 Table LARs of radiation exposure-induced cancer mortality according to age. The additional post-disaster risk was assessed. M: men; W: women. Values in parentheses represent the 2.5–97.5 percentile range.
(PDF)

S7 Table LLEs among patients with diabetes (years). (PDF)

S8 Table LARs of mortality among patients with diabetes. (PDF)

S9 Table LLEs and LARs of mortality due to diabetes by age. The additional post-disaster risk was assessed. Scenario 1: Emerging diabetes occurred only during the first 4 years. Scenario 2: Emerging diabetes occurred during the first 10 years. Premature incidence was considered. Scenario 3: Diabetes prevalence combined the worst cases of scenarios 1 and 2 (only emerging diabetes was considered, and no premature incidence was assumed). The values in parenthesis represent 95% confidence interval.
(PDF)

S10 Table Details of the costs of countermeasures and their defrayers. (PDF)

S11 Table Effects of restricted food distribution on life-years saved. M: men; W: women.
(PDF)

S12 Table Unit and per-capita costs for restricted food distribution. (PDF)

S13 Table Effects of decontamination on life-years saved. (PDF)

S14 Table Effects of whole-body counter tests and interventions on life-years saved. M: men; W: women.
(PDF)

S1 Fig Locations of Minamisoma City and Soma City. (PDF)

S2 Fig Cumulative changes in radiation exposure. 0 y and ≥20 y indicate ages at the time of the disaster. M: men.
(PDF)

S3 Fig **(A) Temporal changes in radiocesium levels in the body and (B) the correlation between measured and predicted radiocesium levels at the second and third measurements.** Individual plots represent residents with high levels of internal contamination after whole-body counter screening. The lines in *A* represent reduction according to the biological half-life, assuming that radiocesium intake stopped 30 d after whole-body counter screening. Under the same assumption, the predicted radiocesium values in *B* were determined according to the biological half-life.
(PDF)
