# Supplementary material for: Additional risk of diabetes exceeds the increased risk of cancer caused by radiation exposure after the Fukushima disaster
Source: PLoS One. 2017 Sep 28;12(9):e0185259. doi: 10.1371/journal.pone.0185259 (PMC5619752; doi:10.1371/journal.pone.0185259)
Supplement: S1 Method — (PDF) [file pone.0185259.s001.pdf]

Supporting Information

Research article

Additional risk of diabetes exceeds the increased risk of cancer caused by radiation exposure after the Fukushima disaster

Michio Murakami,[1,2]* Masaharu Tsubokura,[3,4] Kyoko Ono,[5] Shuhei Nomura,[6,7] Tomoyoshi Oikawa[3]

Affiliations:

[1] Department of Health Risk Communication, Fukushima Medical University School of Medicine, 1 Hikarigaoka, Fukushima, Fukushima, 960-1295, Japan

[2] Radiation Medical Science Center for the Fukushima Health Management Survey, Fukushima Medical University, 1 Hikarigaoka, Fukushima, Fukushima, 960-1295, Japan

[3] Department of Radiation Protection, Minamisoma Municipal General Hospital, 2-54-6 Takami, Haramachi, Minamisoma, Fukushima, 975-0033, Japan

[4] Department of Radiation Protection, Soma Central Hospital, 3-5-18 Okinouchi, Soma, Fukushima, 976-0016, Japan

[5] Research Institute of Science for Safety and Sustainability, National Institute of Advanced Industrial Science and Technology (AIST), 16-1, Onogawa, Tsukuba, Ibaraki, 305–8569, Japan

[6] Department of Epidemiology and Biostatistics, School of Public Health, Imperial College London, Norfolk Place, London W2 1PG, United Kingdom

[7] Department of Global Health Policy, Graduate School of Medicine, The University of Tokyo, 7-3-1, Hongo, Bunkyo, Tokyo 113-0033, Japan

*Corresponding author Email: michio@fmu.ac.jp

S1 Methods

Estimation of additional doses

The additional effective dose from external exposure was separated into two phases: the first 4 months (March 11–July 11, 2011) and thereafter. The effective dose for the first 4 months was determined from average values (excluding radiation workers) in the Soso district (0.8 mSv) [1]. The external exposure from July 12, 2011 and thereafter was estimated using the assumed ambient dose equivalents of 1.08 μSv/h before decontamination on July 1, 2012 and 0.55 μSv/h after decontamination, which were calculated from the average of values from outside 109 residences in contaminated areas of Minamisoma [2]. Temporal changes in effective doses before and after the decontamination were estimated only from physical decay [3] and the radiocesium composition after the accident in 2011 (radiocesium composition, ^{134}Cs:^{137}Cs = 1:1; contribution to additional effective dose, ^{134}Cs:^{137}Cs = 0.73:0.27 on August 23, 2011 [4]). We did not consider weathering effects and can therefore regard the doses as conservative. The additional effective dose from external exposure (D_{ee}) was estimated as:

$$D_{ee} = a_f \times D_{eef} + \int(\mathrm{ADR} \times k - \mathrm{BGR})dt \times \mathrm{RF} \times C_{age} \qquad (1)$$

where a_f = 1 during the first 4 months and 0 thereafter, D_{eef} is the additional effective dose from external exposure during the first 4 months, ADR is the ambient dose equivalent rate, k converts the ADR to an effective dose (0.6 [5]), BGR is the background rate (0.03 μSv/h [5]), RF is the reduction factor, and C_{age} is the correction factor for children (<16 y: $C_{age} = -0.0144 \times \mathrm{age} + 1.27$; ≥16 y: C_{age} = 1 [5]). The RF was set at 0.45, or the average of 0.3 among children [6] and 0.6 in the whole population in a conservative scenario [7]; this value agreed with the value of 0.46 estimated from shielding factors and actual indoor occupancy factors [8].

Doses from inhalation were considered only in the first year, as in a previous study [7]. These doses were reported to be negligible during the second year [9]. Doses were estimated as:

$$D_{ih} = A_{Cs\text{-}137} \times I_i \times \sum_m (A_m/A_{Cs\text{-}137})/V_{bm} \times d_{mi} \qquad (2)$$

where D_{ih} is the additional effective dose from inhalation for age group i [mSv], I_i is the breathing rate of age group i [$m^3\ s^{-1}$], m is the radionuclide (^{131}I, ^{132}I, ^{132}Te, ^{134}Cs, ^{137}Cs), $A_{Cs\text{-}137}$ or A_m is the surface activity density of ^{137}Cs or radionuclide m on the ground [Bq m^{-2}], V_{bm} is the bulk deposition velocity of radionuclide m [m s^{-1}], and d_{mi} is the effective dose inhalation coefficient for radionuclide m and age group i [mSv Bq^{-1}]. The median of the values measured in Minamisoma [10] was used for $A_{Cs\text{-}137}$ to maintain consistency with the external exposure estimates.

Doses from ingestion were assumed to be similar to those in Fukushima City (~50 km from the Fukushima Daiichi NPS) [11]. The doses from ingestion during the first year included ^{131}I, ^{134}Cs and ^{137}Cs, whereas those during the second year and thereafter were

calculated from the doses of ^{134}Cs and ^{137}Cs in March 2012 and from physical decay. The doses from ingestion in Minamisoma and Soma might have been slightly higher than those in Fukushima but were minor relative to the external exposure and inhalation [11,12]. Therefore, this difference would not influence the results.

LLE due to radiation-induced cancer

Ages of 0, 5, 10, 20, 30, 40, 50, 60, 70, and 80 years at the time of the disaster were used to represent ages 0, 1–9, 10–19, 20–29, 30–39, 40–49, 50–59, 60–69, 70–79, and ≥80 years, respectively. We used the mortality risk models for all solid cancers [13] and leukemia [14,15], which were based on the recent Life Span Study cohort of Hiroshima and Nagasaki atomic bomb survivors. A previous study [13] showed that a linear–quadratic dose–response model yielded a better fit in the range of <2 Gy, whereas a linear non-threshold model provided a better fit without range limitation. Therefore, we used the linear–quadratic dose–response model. Organ (colon and bone marrow) doses were calculated from the additional effective doses and from the ratio of the organ dose to effective dose [16]. The excess relative risk (ERR) model for the mortality risk of all solid cancers was:

$$\mathrm{ERR}\,(D, e, a, g) = (\alpha \times D + \beta \times D^2) \times (1 + t \times g) \times \exp[g_e \times (e - 30) + g_a \times \ln(a/70)] \quad (3)$$

where D is the organ dose (Sv), e is the age at exposure, a is the age attained, g is sex, $\alpha = 0.22$, $\beta = 0.18$, $t = 0.29$, $g = -1$ for men and 1 for women, $g_e = -0.034$, and $g_a = -0.89$.

The ERR model for the mortality risk model of leukemia was:

$$\mathrm{ERR}\,(D, a) = (\alpha \times D + \beta \times D^2) \times \exp[\gamma \times \ln[a/50]] \quad (4)$$

where $\alpha = 1.612$, $\beta = 1.551$, and $\gamma = -1.634$.

The minimum latency periods were set at 5 years for all solid cancers and 2 years for leukemia. The mortality rates for all solid cancers and leukemia were determined from the age- and sex-stratified all-cause mortality in Japan [17]. Based on these estimated additional age-specific mortality rates, the LARs of mortality and LLEs were calculated from the survival probabilities of Japanese men and women [18] **(S5** and **S6 Tables**).

LLE due to diabetes

The ages of 40, 50, 60, and 70 years in 2011 were used to represent the age groups 40–49, 50–59, 60–69 and 70–79 years, respectively. Assuming that the prevalence of diabetes would not improve and that the time-changes in diabetes prevalence consequent to the increased mortality rates would be negligible, the additional incidence of diabetes AD was estimated as:

$$\mathrm{AD}(x, t) = P_{\mathrm{ad}}(x, t) - P_{\mathrm{bl}}(x, t) - (P_{\mathrm{ad}}(x, t\text{-}1) - P_{\mathrm{bl}}(x, t\text{-}1)) \quad (5)$$

where x is the age, t is the time stage (1: March 11, 2011–March 10, 2015; 2: March 11, 2015–March 10, 2021; 3: March 11, 2021–), P_{ad} is the diabetes prevalence ratio after the disaster, P_{bl} is the diabetes prevalence ratio at baseline (same as before the disaster), and $P_{\mathrm{ad}}(x,$

$0) - P_{bl}(x, 0)=0$.

The cohort study [19] from which data in this study were obtained was initiated in 1990 and ended in 2010 (median follow-up: 17.8 years). The ages of participants ranged from 40 to 69 years, and a total of 99 584 participants (men: 46 017; women: 53 567) were included. We excluded participants with any of the following conditions at baseline: cardiovascular disease, chronic liver disease, kidney disease, any cancer, or a body mass index (BMI) of <14 or >40. Here, a history of diabetes (i.e., physician diagnosis at any point) or the current use of anti-diabetes drugs was used to indicate diabetes.

The adjusted HR for all-cause mortality related to diabetes depended on the period of diagnosis: among men, before baseline = 1.59, between baseline and the 5-year survey = 1.20, between the 5- and 10-year surveys = 1.22; among women, before baseline = 2.00, between baseline and the 5-year survey = 1.55, and between the 5- and 10-year surveys = 1.45. Because the median follow-up period was 17.8 years, we classified HRs into ≤15 years and >15 years after the incidence of diabetes. In summary, the following HRs were used: for men, ≤15 years after the incidence of diabetes, 1.20 and >15 years after, 1.59; for women, ≤15 years after the incidence of diabetes, 1.45 and >15 years after, 2.00. However, these values may lead to an underestimation of risk (see details in "Sources of uncertainty").

Additional diabetes-induced mortality rates (Ce) were estimated as:

$$\mathrm{Ce}(x) = M(x) \times R(x) \times (\mathrm{HR} - 1) \qquad (6)$$

where $M(x)$ is the all-cause mortality rate and $R(x)$ is the ratio of all-cause mortality among those without diabetes to the total, calculated as:

$$R(x) = (1 - P(x))/(1 - P(x) + P(x) \times \mathrm{HR}) \qquad (7)$$

where $P(x)$ is the prevalence of diabetes.

The $R(x)$ was calculated from the $P(x)$ in 2012 in Japan [20] and the HR (1.59 for men, 2.00 for women). The LARs of mortality and LLEs of a patient with diabetes were calculated using the $\mathrm{Ce}(x)$ and the Japanese survival probability (**S7** and **S8 Tables**). The LARs of mortality and LLEs due to diabetes in each scenario were then estimated from the additional incidence of diabetes and the LLEs of patients with diabetes during years 1–4 and 5–10 **(S9 Table**). In Scenario 2, we considered the premature incidence among men in their 40s and 50s to obtain the LLEs (or LARs of mortality) during years1–4, and subtracted the LLEs (or LARs of mortality) estimated from the negative incidence of diabetes during years 5–10 (additional incidence: −3.7% for men in their 40s; −4.1% for men in their 50s) from the LLEs (or LARs of mortality) estimated from the incidence of diabetes during years 1–4.

Costs and effectiveness of countermeasures

The effects of restricted food distribution were estimated from the reduction in the effective dose from dietary sources in Fukushima City, the prefectural capital, during the first year after the disaster (March 21, 2011–March 20, 2012) as reported in a previous study [11],

rather than from the cities of Minamisoma and Soma. Because restricted food distribution was used for market regulation, Fukushima City was considered an appropriate target area. The life-years saved (LYS) were calculated from the reduction in dose for each sex and by age group (0, 5, 10, 20, 30, 40, 50, 60, 70, 80 years) using the above-described models (**S11 Table**). The LYS were then estimated for the whole population on March 1, 2011 [21]. The cost of the restricted food distribution comprised the costs of foods produced but discarded (CF) (i.e., opportunity losses) and of monitoring. The CF was estimated as:

$$\mathrm{CF}_j = \sum_{i,j,k} A_j \times B_{i,j} \times C_{j,k} \times X_{j,k} \qquad (8)$$

where j is the food category (16 categories: rice, dairy products, milk, tea, turnips, spinach, garland chrysanthemum and ging-geng-cai, mustard spinach and non-heading lettuce, heading leafy vegetables, broccoli and cauliflower, kiwifruit, chestnut, bamboo shoots, mushrooms, beef, and wild *ayu*, wild Japanese dace and wild landlocked *masu* salmon [11]), i is the age and sex group, k is the area where food distribution was restricted, A_j is the unit cost of food j (JPY/kg), $B_{i,j}$ is the daily consumption of food j per person i (kg/d), $C_{j,k}$ is the number of days food j distribution was restricted in area k (d), and $X_{j,k}$ is the arrival share (the fraction of food j on the market in Fukushima City that came from area k).

A_j was determined from previous reports [22-25]. The prices for shipping and selling on-cost (39.3%) were not included [22]. The median value of beef was used. The per-capita costs were calculated as 700 JPY (**S12 Table**). The total costs of monitoring foods and tap water were estimated from the unit cost of measurement (10 000 JPY) and the total number of measurements [26-29]. Food measurements were considered to be applicable for the whole Japanese population, and accordingly the total cost per person, 10 JPY, was estimated by dividing the total cost by the total number of people in Japan [30]. For tap water, we counted the number of monitoring tests in Fukushima City and divided the total cost by the total number of people in Fukushima City to yield 40 JPY. The final monitoring cost (50 JPY) was minor when compared with the opportunity loss for foods (700 JPY); in other words, uncertainty in the monitoring cost is unlikely to influence the results. The cost per life-years saved (CPLYS) of the restricted food distribution was 56 million JPY/year, comparable to those for vegetables from March to May 2011 (6.6–240 million JPY/year) and lower than those for rice in 2011 (310–1000 million JPY/year) [22].

The effect of decontamination was estimated under the assumption described in “Estimation of additional doses”, namely a reduction in the ambient dose equivalent from 1.08 to 0.55 μSv/h outside of residences on July 1, 2012 [2]. The doses were considered to decrease with physical decay. The LYS was calculated based on the estimated effective doses from external exposure (eq. 1) (**S13 Table**). The costs of decontamination in Minamisoma and Soma were taken from the literature [31-33]. For Minamisoma, the costs were the median values in scenarios wherein areas with effective doses of >1 mSv/year were decontaminated (details were provided by the author of the sources [31,32]). For Soma, the costs were

estimated from unit prices and the actual number of decontamination events planned to March 2016.

The effects of whole-body counter tests and interventions were estimated from the results of a whole-body counter screening and counseling program conducted at Minamisoma Municipal General Hospital and Hirata Central Hospital (Hirata Village, ~40 km from Fukushima Daiichi NPS) [34]. Although whole-body counter screening was conducted in 2011, the effects of this screening were not clear because the dose reduction was attributed mainly to the physical and environmental decay of radiocesium in foods rather than to medical counseling. Therefore, we used the results obtained from March 11, 2012 to March 10, 2013. Of the 30 622 residents who participated in whole-body counter screening, 9 were found to have radiocesium levels exceeding 50 Bq/kg, and their effective doses from internal exposure to ^{134}Cs and ^{137}Cs ranged from 0.14 to 0.97 mSv/year. After counseling to reduce the intake of locally grown foods (generally, 30 d after the measurement), the 8 residents who remained in the study exhibited dynamic reductions in radiocesium levels (one resident dropped out from subsequent screenings) (**S3 Figure**). The ^{137}Cs levels measured at the second and third screenings agreed well with estimates based on the biological half-life [35] and the assumption that radiocesium intake stopped 30 d after the whole-body counter screening. Therefore, we considered the effects of whole-body counter tests and interventions as the differences in the lifetime effective doses from internal exposure between the continuous intake of radiocesium from contaminated foods (0.14–0.97 mSv/year at the first measurement) and the average intake of foods in Fukushima Prefecture (0.0022 mSv/year, measured using food duplicate methods in March–May 2012 [36]). The ratio of ^{134}Cs:^{137}Cs on March 11, 2011 was assumed to be 1:1, and radiocesium concentrations in foods were assumed to decrease by physical decay. The dose coefficient was taken from [37]. The LYS was similarly calculated using dose reduction (**S14 Table**). The costs for whole-body counter screening comprised instruments, consultation, travel, and time. One whole-body counter scanner cost 45.3 million JPY, and four scanners were installed at Minamisoma Municipal General Hospital and Hirata Central Hospital. The useful life span was set at 10 years, and the costs for 1 year were estimated. A discount rate was not considered. One measurement and consultation cost 5000 JPY. The travel costs for one measurement were estimated using the unit price of gasoline (120 JPY/L), travel distance (20 km for Minamisoma; 60 km for Hirata), and gasoline consumption rate (10 km/L). The time was calculated from the unit value (20 JPY/min) and the travel speed (30 km/h for Minamisoma; 50 km/h for Hirata). The total number of individuals was 30 622 (cumulative number: 9969 in Minamisoma and 20 668 in Hirata; some participated more than once).

As a countermeasure against diabetes, we considered health checkups and metformin therapy for diabetes, as metformin therapy was shown to be effective for overweight patients with Type 2 diabetes (>120% ideal body weight; body mass index (BMI) = 25.6 kg/m^2) in a

randomized controlled trial that used the outcome of all-cause mortality in the UK (median duration: 10.7 years) [38,39]. Although empagliflozin was recently reported to reduce mortality associated with cardiovascular causes among for patients with Type 2 diabetes [40], we did not consider the cost-effectiveness of this drug because of a lack of efficient practical applications. Although we needed to estimate the differences in LYS between therapy and no therapy, data were not available for the latter. Because metformin therapy for overweight patients increased the LYS per patient by 1.0 year when compared with other conventional therapies, we expected that it would also increase the LYS per patient by >1.0 year when compared with no therapy. The LYS for the whole population was estimated from the prevalence of diabetes among the overweight (BMI >25.6 kg/m^2) in Minamisoma and Soma during the period of 2012–2014 (men: 5.6%; women: 3.5%). The costs comprised health checkups and the total costs associated with therapy and complications. The costs for health checkups applied to all participants, as all were screened for diabetes. The unit price of a health checkup is 8000 JPY/person, and we assumed that individuals would undergo 35 total health checkups (annually in the age range of 40–74 years). The total costs of metformin therapy and complications, 7883 GBP/patient (≈ 1.26 million JPY/patient on the basis of 160 JPY/GBP), were obtained from a UK study and were lower than those obtained for other conventional therapies (8165 GBP/patient ≈ 1.31 million JPY/patient) [39]. The cost of health checkups was greatly overestimated because these are not performed merely to identify overweight patients with diabetes. Overall, the CPLYSs for health checkups and metformin therapy for diabetes were overestimated by up to 7.4 million JPY/year.

References

1. Ishikawa T, Yasumura S, Ozasa K, Kobashi G, Yasuda H, Miyazaki M, et al. (2015) The Fukushima Health Management Survey: Estimation of external doses to residents in Fukushima Prefecture. Sci Rep 5: 12712.
2. Minamisoma City (2013) http://nuce.aesj.or.jp/_media/ss:ss29:08_%E5%8D%97%E7%9B%B8%E9%A6%AC%E5%B8%82%E3%81%AE%E9%99%A4%E6%9F%93%E7%8A%B6%E6%B3%81%E3%81%AB%E3%81%A4%E3%81%84%E3%81%A6.pdf. Accessed: [in Japanese]
3. ICRP (2008) Nuclear decay data for dosimetric calculations. ICRP Publication 107. Ann ICRP 38.
4. Nuclear Emergency Response Headquarters (2011) Prediction of future air dose rate from current air dose. [in Japanese]
5. Akahane K, Yonai S, Fukuda S, Miyahara N, Yasuda H, Iwaoka K, et al. (2013) NIRS external dose estimation system for Fukushima residents after the Fukushima Dai-ichi NPP accident. Sci Rep 3: 1670.

6. Nomura S, Tsubokura M, Hayano R, Furutani T, Yoneoka D, Kami M, et al. (2015) Comparison between direct measurements and modeled estimates of external radiation exposure among school children 18 to 30 months after the Fukushima nuclear accident in Japan. Environ Sci Technol 49: 1009-1016.
7. World Health Organization (2012) Preliminary dose estimation from the nuclear accident after the 2011 Great East Japan Earthquake and Tsunami.
8. Bedwell P, Mortimer K, Wellings J, Sherwood J, Leadbetter SJ, Haywood SM, et al. (2015) An assessment of the doses received by members of the public in Japan following the nuclear accident at Fukushima Daiichi nuclear power plant. J Radiol Prot 35: 869-890.
9. Harada KH, Niisoe T, Imanaka M, Takahashi T, Amako K, Fujii Y, et al. (2014) Radiation dose rates now and in the future for residents neighboring restricted areas of the Fukushima Daiichi nuclear power plant. P Natl Acad Sci USA 111: E914-E923.
10. Ministry of Education Culture Sports Science and Technology (2011) Results of radionuclides (^{134}Cs and ^{137}Cs) in soils: http://www.mext.go.jp/b_menu/shingi/chousa/gijyutu/017/shiryo/__icsFiles/afieldfile/2011/09/02/1310688_1.pdf. Accessed: Sep. 2014 [in Japanese]
11. Murakami M, Oki T (2014) Estimated dietary intake of radionuclides and health risks for the citizens of Fukushima City, Tokyo, and Osaka after the 2011 nuclear accident. PLos One 9: e112791.
12. Tsubokura M, Kato S, Morita T, Nomura S, Kami M, Sakaihara K, et al. (2015) Assessment of the annual additional effective doses amongst Minamisoma children during the second year after the Fukushima Daiichi nuclear power plant disaster. PLos One 10: e0129114.
13. Ozasa K, Shimizu Y, Suyama A, Kasagi F, Soda M, Grant EJ, et al. (2012) Studies of the mortality of atomic bomb survivors, Report 14, 1950-2003: An overview of cancer and noncancer diseases. Radiat Res 177: 229-243.
14. United Nations Scientific Committee on the Effects of Atomic Radiation (2006) Report of the United Nations Scientific Committee on the Effects of Atomic Radiation. UNSCEAR 2006 report. New York.
15. Journy N, Ancelet S, Rehel J-L, Mezzarobba M, Aubert B, Laurier D, et al. (2014) Predicted cancer risks induced by computed tomography examinations during childhood, by a quantitative risk assessment approach. Radiat Environ Bioph 53: 39-54.
16. World Health Organization (2013) Health risk assessment from the nuclear accident after the 2011 Great East Japan Earthquake and Tsunami based on a preliminary dose estimation.

17. Statistics Bureau of Japan (2011) General mortality in 2010: http://www.e-stat.go.jp/SG1/estat/ListE.do?lid=000001101825. Accessed: Mar. 2014
18. Ministry of Health Labour and Welfare (2012) The 21st life tables: http://www.mhlw.go.jp/toukei/saikin/hw/life/21th/index.html. Accessed: Sep. 2014 [in Japanese]
19. Kato M, Noda M, Mizoue T, Goto A, Takahashi Y, Matsushita Y, et al. (2015) Diagnosed diabetes and premature death among middle-aged Japanese: results from a large-scale population-based cohort study in Japan (JPHC study). BMJ open 5: e007736.
20. Ministry of Health Labour and Welfare (2013) National Health and Nutrition Survey 2012. [in Japanese]
21. Fukushima Prefecture (2016) https://www.pref.fukushima.lg.jp/sec/11045b/15859.html. Accessed: July, 2016 [in Japanese]
22. Oka T (2014) Cost-benefit analysis of the regulation of food contamination with radioactive caesiums within a year after the Fukushima accident: The cases of vegetables and rice. Jpn J Risk Anal 24: 101-110. [in Japanese]
23. Ministry of Agriculture Forestry and Fisheries (2012) Statistics of Agricultural prices 2012: http://www.e-stat.go.jp/SG1/estat/List.do?lid=000001083123. Accessed: [in Japanese]
24. Fukushima City Central Wholesale Market (2016) http://www.city.fukushima.fukushima.jp/soshiki/24/1030.html. Accessed: July 2016 [in Japanese]
25. Oka T (2012) Risk/benefit analysis of the regulation of foodstuffs contaminated with radioactive substances. Soc Environ Econ Policy Stud. [in Japanese]
26. Ministry of Agriculture Forestry and Fisheries (2014) http://www.maff.go.jp/j/kanbo/joho/saigai/s_chosa/kome_kinkyu_chosa.html. Accessed: July, 2016 [in Japanese]
27. Ministry of Health Labour and Welfare (2016) http://www.mhlw.go.jp/stf/houdou/2r98520000029prx.html. Accessed: July 2016 [in Japanese]
28. Fukushima Water Supply Authority (2016) http://www.f-wsa.jp/suishistsu/nenpou/23nennpou.html. Accessed: July 2016 [in Japanese]
29. Ministry of Health Labour and Welfare (2016a) http://www.mhlw.go.jp/stf/seisakunitsuite/bunya/topics/bukyoku/kenkou/suido/kentoukai/houshasei_monitoring.html. Accessed: July 2016 [in Japanese]
30. Statistics Bureau of Japan (2011) Population Estimates by Age (5-Year Age Group) and Sex -Total population,Japanese population:

http://www.e-stat.go.jp/SG1/estat/List.do?bid=000001036209&cycode=0. Accessed: July 2016 [in Japanese]

31. Yasutaka T, Naito W, Nakanishi J (2013) Cost and effectiveness of decontamination strategies in radiation contaminated areas in Fukushima in regard to external radiation dose. PLos One 8: e75308.
32. Yasutaka T, Naito W (2016) Assessing cost and effectiveness of radiation decontamination in Fukushima Prefecture, Japan. J Environ Radioact 151 Pt 2: 512-520.
33. Environmental Remediation Ministry of Environment (2016) http://josen.env.go.jp/zone/index.html. Accessed: June 2016 [in Japanese]
34. Tsubokura M, Kato S, Nomura S, Gilmour S, Nihei M, Sakuma Y, et al. (2014) Reduction of high levels of internal radio-contamination by dietary intervention in residents of areas affected by the Fukushima Daiichi nuclear plant disaster: A case series. PLos One 9: e100302.
35. ICRP (1993) Age-dependent doses to members of the public from intake of radionuclides: Part 2 ingestion dose coefficients. ICRP Publication 67, Ann. ICRP 23, 3-4.
36. Ministry of Health Labour and Welfare (2013) http://www.mhlw.go.jp/stf/houdou/2r9852000002wyf2-att/2r9852000002wyjc.pdf. Accessed: July, 2016 [in Japanese]
37. ICRP (1996) Age-dependent doses to the members of the public from intake of radionuclides - Part 5 compilation of ingestion and inhalation coefficients. Ann ICRP 26.
38. UK Prospective Diabetes Study (UKPDS) Group (1998) Effect of intensive blood-glucose control with metformin on complications in overweight patients with type 2 diabetes (UKPDS 34). Lancet 352: 854-865.
39. Clarke P, Gray A, Adler A, Stevens R, Raikou M, Cull C, et al. (2001) Cost-effectiveness analysis of intensive blood-glucose control with metformin in overweight patients with type II diabetes (UKPDS No. 51). Diabetologia 44: 298-304.
40. Zinman B, Wanner C, Lachin JM, Fitchett D, Bluhmki E, Hantel S, et al. (2015) Empagliflozin, cardiovascular outcomes, and mortality in Type 2 diabetes. N Engl J Med 373: 2117-2128.
