# Supplementary material for: Additional risk of diabetes exceeds the increased risk of cancer caused by radiation exposure after the Fukushima disaster
Source: PLoS One. 2017 Sep 28;12(9):e0185259. doi: 10.1371/journal.pone.0185259 (PMC5619752; doi:10.1371/journal.pone.0185259)
Supplement: S1 Table — The values in parenthesis represent 95% confidence interval. (PDF) [file pone.0185259.s002.pdf]

S1 Table.

Numbers of male patients with diabetes and total participants, and the prevalence before and after the disaster. The values in parenthesis represent 95% confidence interval.

	40–43	44–49	50–53	54–59	60–63	64–69	70–
Patients with diabetes/total participants							
2008	1/36	7/112	1/89	36/313	44/355	86/800	12/186
2009	1/28	6/100	4/102	33/258	43/383	84/776	36/310
2010	2/44	2/90	5/92	28/250	51/412	94/789	54/470
2011	0/16	1/47	4/63	15/124	23/168	48/420	56/420
2012	2/16	2/57	4/79	19/171	32/223	84/652	100/640
2013	0/7	2/54	8/74	19/139	28/194	99/626	122/641
2014	0/4	3/40	6/67	15/117	27/169	93/560	114/667
Before the disaster	4/108	15/302	10/283	97/821	138/1150	264/2365	102/966
After the disaster [a]	2/27	7/151	18/220	53/427	87/586	276/1838	336/1948
Prevalence							
Before the disaster	3.7% (0.1%–7.3%)	5.0% (2.5%–7.4%)	3.5% (1.4%–5.7%)	11.8% (9.6%–14.0%)	12.0% (10.1%–13.9%)	11.2% (9.9%–12.4%)	10.6% (8.6%–12.5%)
After the disaster [a]	7.4% (−2.5%–17.3%)	4.6% (1.3%–8.0%)	8.2% (4.6%–11.8%)	12.4% (9.3%–15.5%)	14.8% (12.0%–17.7%)	15.0% (13.4%–16.6%)	17.2% (15.6%–18.9%)
Δ	3.7% (−6.8%–14.2%)	−0.3% (−4.5%–3.8%)	4.6% (0.4%–8.9%)	0.6% (−3.2%–4.4%)	2.8% (−0.6%–6.3%)	3.9% (1.8%–5.9%)	6.7% (4.1%–9.3%)

[a] Results in 2011 were excluded owing to potential biases of participants.
