# Supplementary material for: Additional risk of diabetes exceeds the increased risk of cancer caused by radiation exposure after the Fukushima disaster
Source: PLoS One. 2017 Sep 28;12(9):e0185259. doi: 10.1371/journal.pone.0185259 (PMC5619752; doi:10.1371/journal.pone.0185259)
Supplement: S2 Table — The values in parenthesis represent 95% confidence interval. (PDF) [file pone.0185259.s003.pdf]

S2 Table.

Numbers of female patients with diabetes and total participants, and the prevalence before and after the disaster. The values in parenthesis represent 95% confidence interval.

	40–43	44–49	50–53	54–59	60–63	64–69	70–
Patients with diabetes/total participants							
2008	2/54	2/117	4/158	24/530	31/627	74/1117	14/226
2009	0/40	2/111	5/149	20/468	34/702	69/1068	31/383
2010	1/54	1/99	4/133	14/439	40/743	62/1073	55/583
2011	0/20	1/50	4/81	12/213	18/332	27/588	37/518
2012	0/20	1/77	3/80	16/270	28/428	71/985	69/798
2013	0/9	3/71	3/68	18/227	29/397	95/986	83/825
2014	0/1	2/60	2/58	13/205	20/346	87/953	101/920
Before the disaster	3/148	5/327	13/440	58/1437	105/2072	205/3258	100/1192
After the disaster [a]	0/30	6/208	8/206	47/702	77/1171	253/2924	253/2543
Prevalence							
Before the disaster	2.0% (−0.2%–4.3%)	1.5% (0.2%–2.9%)	3.0% (1.4%–4.5%)	4.0% (3.0%–5.1%)	5.1% (4.1%–6.0%)	6.3% (5.5%–7.1%)	8.4% (6.8%–10.0%)
After the disaster [a]	0.0% (0.0%–0.0%)	2.9% (0.6%–5.2%)	3.9% (1.2%–6.5%)	6.7% (4.8%–8.5%)	6.6% (5.2%–8.0%)	8.7% (7.6%–9.7%)	9.9% (8.8%–11.1%)
Δ	−2.0% (−4.3%–0.2%)	1.4% (−1.3%–4.0%)	0.9% (−2.1%–4.0%)	2.7% (0.5%–4.8%)	1.5% (−0.2%–3.2%)	2.4% (1.0%–3.7%)	1.6% (−0.4%–3.5%)

[a] Results in 2011 were excluded owing to potential biases of participants.
