# Supplementary material for: Additional risk of diabetes exceeds the increased risk of cancer caused by radiation exposure after the Fukushima disaster
Source: PLoS One. 2017 Sep 28;12(9):e0185259. doi: 10.1371/journal.pone.0185259 (PMC5619752; doi:10.1371/journal.pone.0185259)
Supplement: S3 Table — Scenario 1: Emerging diabetes occurred only during the first 4 years. Scenario 2: Emerging diabetes occurred during the first 10 years. Premature incidence was considered. Scenario 3: Diabetes prevalence combined the worst cases of scenarios 1 and 2 (only emerging diabetes was considered, and no premature incidence was assumed). The values in parenthesis represent 95% confidence interval. (PDF) [file pone.0185259.s004.pdf]

S3 Table.

Prevalence and additional incidence of diabetes at baseline and in each scenario among men. Scenario 1: Emerging diabetes occurred only during the first 4 years. Scenario 2: Emerging diabetes occurred during the first 10 years. Premature incidence was considered. Scenario 3: Diabetes prevalence combined the worst cases of scenarios 1 and 2 (only emerging diabetes was considered, and no premature incidence was assumed). The values in parenthesis represent 95% confidence interval.

		Years 1–4	Years 5–10	Years 11–
Age at the disaster				
40	Prevalence			
	Baseline	3.7% (0.1%–7.3%)	5.0% (2.5%–7.4%)	3.5%
	Scenario 1	7.4% (−2.5%–17.3%)	8.7% (−2.1%–19.5%)	7.2%
	Scenario 2	7.4% (−2.5%–17.3%)	4.6% (1.3%–8.0%)	3.2%
	Scenario 3	7.4% (−2.5%–17.3%)	8.7% (−2.1%–19.5%)	7.2%
	Additional incidence			
	Scenario 1	3.7% (−6.8%–14.2%)	0.0% (–)	0.0%
	Scenario 2	3.7% (−6.8%–14.2%)[a]	−4.0% (−15.3%–7.3%)[a]	0.0%
	Scenario 3	3.7% (−6.8%–14.2%)	0.0% (–)	0.0%
50	Prevalence			
	Baseline	3.5% (1.4%–5.7%)	11.8% (9.6%–14.0%)	12.0%
	Scenario 1	8.2% (4.6%–11.8%)	16.5% (11.7%–21.2%)	16.6%
	Scenario 2	8.2% (4.6%–11.8%)	12.4% (9.3%–15.5%)	12.6%
	Scenario 3	8.2% (4.6%–11.8%)	16.5% (11.7%–21.2%)	16.6%
	Additional incidence			
	Scenario 1	4.6% (0.4%–8.9%)	0.0% (–)	0.0%
	Scenario 2	4.6% (0.4%–8.9%)[a]	−4.1% (−9.7%–1.6%)[a]	0.0%
	Scenario 3	4.6% (0.4%–8.9%)	0.0% (–)	0.0%
60	Prevalence			
	Baseline	12.0% (10.1%–13.9%)	11.2% (9.9%–12.4%)	10.6%
	Scenario 1	14.8% (12.0%–17.7%)	14.0% (10.3%–17.7%)	13.4%
	Scenario 2	14.8% (12.0%–17.7%)	15.0% (13.4%–16.6%)	14.4%
	Scenario 3	14.8% (12.0%–17.7%)	15.0% (13.4%–16.6%)	14.4%
	Additional incidence			
	Scenario 1	2.8% (−0.6%–6.3%)	0.0% (–)	0.0%
	Scenario 2	2.8% (−0.6%–6.3%)	1.0% (−3.0%–5.0%)	0.0%
	Scenario 3	2.8% (−0.6%–6.3%)	1.0% (−3.0%–5.0%)	0.0%
70	Prevalence			
	Baseline	10.6% (8.6%–12.5%)	10.6% (8.6%–12.5%)	10.6%
	Scenario 1	17.2% (15.6%–18.9%)	17.2% (15.6%–18.9%)	17.2%
	Scenario 2	17.2% (15.6%–18.9%)	17.2% (15.6%–18.9%)	17.2%
	Scenario 3	17.2% (15.6%–18.9%)	17.2% (15.6%–18.9%)	17.2%
	Additional incidence			
	Scenario 1	6.7% (4.1%–9.3%)	0.0% (–)	0.0%
	Scenario 2	6.7% (4.1%–9.3%)	0.0% (–)	0.0%
	Scenario 3	6.7% (4.1%–9.3%)	0.0% (–)	0.0%

[a] Increase of diabetes prevalence during the first 4 years was regarded as premature incidence.
