# Supplementary material for: Additional risk of diabetes exceeds the increased risk of cancer caused by radiation exposure after the Fukushima disaster
Source: PLoS One. 2017 Sep 28;12(9):e0185259. doi: 10.1371/journal.pone.0185259 (PMC5619752; doi:10.1371/journal.pone.0185259)
Supplement: S4 Table — Scenario 1: Emerging diabetes occurred only during the first 4 years. Scenario 2: Emerging diabetes occurred during the first 10 years. Premature incidence was considered. Scenario 3: Diabetes prevalence combined the worst cases of scenarios 1 and 2 (only emerging diabetes was considered, and no premature incidence was assumed). The values in parenthesis represent 95% confidence interval. (PDF) [file pone.0185259.s005.pdf]

S4 Table.

Prevalence and additional incidence of diabetes at baseline and in each scenario among women. Scenario 1: Emerging diabetes occurred only during the first 4 years. Scenario 2: Emerging diabetes occurred during the first 10 years. Premature incidence was considered. Scenario 3: Diabetes prevalence combined the worst cases of scenarios 1 and 2 (only emerging diabetes was considered, and no premature incidence was assumed). The values in parenthesis represent 95% confidence interval.

		Years 1–4	Years 5–10	Years 11–
Age at the disaster				
40	Prevalence			
	Baseline	2.0% (−0.2%–4.3%)	1.5% (0.2%–2.9%)	3.0%
	Scenario 1	0.0% (0.0%–0.0%)	−0.5% (−3.1%–2.1%)	0.9%
	Scenario 2	0.0% (0.0%–0.0%)	2.9% (0.6%–5.2%)	4.3%
	Scenario 3	0.0% (0.0%–0.0%)	2.9% (0.6%–5.2%)	4.3%
	Additional incidence			
	Scenario 1	−2.0% (−4.3%–0.2%)	0.0% (–)	0.0%
	Scenario 2	−2.0% (−4.3%–0.2%)	3.4% (−0.1%–6.9%)	0.0%
	Scenario 3	−2.0% (−4.3%–0.2%)	3.4% (−0.1%–6.9%)	0.0%
50	Prevalence			
	Baseline	3.0% (1.4%–4.5%)	4.0% (3.0%–5.1%)	5.1%
	Scenario 1	3.9% (1.2%–6.5%)	5.0% (1.7%–8.2%)	6.0%
	Scenario 2	3.9% (1.2%–6.5%)	6.7% (4.8%–8.5%)	7.7%
	Scenario 3	3.9% (1.2%–6.5%)	6.7% (4.8%–8.5%)	7.7%
	Additional incidence			
	Scenario 1	0.9% (−2.1%–4.0%)	0.0% (–)	0.0%
	Scenario 2	0.9% (−2.1%–4.0%)	1.7% (−2.0%–5.5%)	0.0%
	Scenario 3	0.9% (–2.1%–4.0%)	1.7% (−2.0%–5.5%)	0.0%
60	Prevalence			
	Baseline	5.1% (4.1%–6.0%)	6.3% (5.5%–7.1%)	8.4%
	Scenario 1	6.6% (5.2%–8.0%)	7.8% (5.9%–9.7%)	9.9%
	Scenario 2	6.6% (5.2%–8.0%)	8.7% (7.6%–9.7%)	10.7%
	Scenario 3	6.6% (5.2%–8.0%)	8.7% (7.6%–9.7%)	10.7%
	Additional incidence			
	Scenario 1	1.5% (−0.2%–3.2%)	0.0% (–)	0.0%
	Scenario 2	1.5% (−0.2%–3.2%)	0.9% (−1.3%–3.0%)	0.0%
	Scenario 3	1.5% (−0.2%–3.2%)	0.9% (−1.3%–3.0%)	0.0%
70	Prevalence			
	Baseline	8.4% (6.8%–10.0%)	8.4% (6.8%–10.0%)	8.4%
	Scenario 1	9.9% (8.8%–11.1%)	9.9% (8.8%–11.1%)	9.9%
	Scenario 2	9.9% (8.8%–11.1%)	9.9% (8.8%–11.1%)	9.9%
	Scenario 3	9.9% (8.8%–11.1%)	9.9% (8.8%–11.1%)	9.9%
	Additional incidence			
	Scenario 1	1.6% (−0.4%–3.5%)	0.0% (–)	0.0%
	Scenario 2	1.6% (−0.4%–3.5%)	0.0% (–)	0.0%
	Scenario 3	1.6% (−0.4%–3.5%)	0.0% (–)	0.0%
