# Supplementary material for: Additional risk of diabetes exceeds the increased risk of cancer caused by radiation exposure after the Fukushima disaster
Source: PLoS One. 2017 Sep 28;12(9):e0185259. doi: 10.1371/journal.pone.0185259 (PMC5619752; doi:10.1371/journal.pone.0185259)
Supplement: S5 Table — The additional post-disaster risk was assessed. M: men; W: women. Values in parentheses represent the 2.5–97.5 percentile range. (PDF) [file pone.0185259.s006.pdf]

**S5 Table.**

LLEs among age groups as a result of radiation exposure-induced cancer. The additional post-disaster risk was assessed. M: men; W: women. Values in parentheses represent the 2.5–97.5 percentile range.

| Age at the disaster | Population (persons) | LLEs as a result of radiation exposure-induced cancer in each stage ($10^{-2}$ years) | | | |
|---|---|---|---|---|---|
| | | Years 1–4 | Years 5–10 | Years 11– | Total |
| 0 (M) | 458 | 1.42 (0.77–2.46) | 0.47 (0.24–0.87) | 0.66 (0.33–1.21) | 2.55 (1.71–3.74) |
| 0 (W) | 441 | 1.87 (0.96–3.39) | 0.66 (0.32–1.25) | 0.90 (0.43–1.70) | 3.44 (2.24–5.16) |
| 5 (M) | 4436 | 1.01 (0.51–1.85) | 0.38 (0.19–0.69) | 0.52 (0.27–0.95) | 1.91 (1.27–2.86) |
| 5 (W) | 4261 | 1.42 (0.69–2.68) | 0.52 (0.25–0.99) | 0.71 (0.34–1.35) | 2.66 (1.70–4.08) |
| 10 (M) | 5268 | 0.79 (0.40–1.45) | 0.28 (0.14–0.52) | 0.43 (0.22–0.78) | 1.50 (0.99–2.25) |
| 10 (W) | 4972 | 1.10 (0.53–2.09) | 0.39 (0.19–0.75) | 0.58 (0.28–1.10) | 2.07 (1.32–3.18) |
| 20 (M) | 4933 | 0.47 (0.23–0.87) | 0.19 (0.09–0.35) | 0.29 (0.15–0.52) | 0.95 (0.62–1.41) |
| 20 (W) | 4470 | 0.66 (0.31–1.26) | 0.26 (0.12–0.50) | 0.37 (0.18–0.69) | 1.29 (0.82–1.97) |
| 30 (M) | 6914 | 0.34 (0.17–0.62) | 0.14 (0.07–0.25) | 0.18 (0.10–0.32) | 0.65 (0.43–0.97) |
| 30 (W) | 6494 | 0.46 (0.22–0.87) | 0.18 (0.09–0.34) | 0.22 (0.11–0.41) | 0.86 (0.55–1.32) |
| 40 (M) | 6112 | 0.24 (0.12–0.43) | 0.09 (0.05–0.17) | 0.10 (0.05–0.17) | 0.43 (0.29–0.65) |
| 40 (W) | 5927 | 0.31 (0.15–0.58) | 0.12 (0.06–0.22) | 0.12 (0.06–0.22) | 0.54 (0.35–0.84) |
| 50 (M) | 7765 | 0.16 (0.09–0.29) | 0.058 (0.03–0.10) | 0.045 (0.02–0.08) | 0.27 (0.18–0.40) |
| 50 (W) | 7810 | 0.19 (0.10–0.36) | 0.068 (0.03–0.12) | 0.056 (0.03–0.10) | 0.32 (0.20–0.49) |
| 60 (M) | 7931 | 0.094 (0.051–0.164) | 0.030 (0.016–0.051) | 0.015 (0.008–0.025) | 0.14 (0.09–0.21) |
| 60 (W) | 7867 | 0.11 (0.05–0.19) | 0.035 (0.02–0.06) | 0.020 (0.01–0.04) | 0.16 (0.10–0.25) |
| 70 (M) | 5392 | 0.043 (0.024–0.074) | 0.010 (0.006–0.017) | 0.003 (0.001–0.004) | 0.056 (0.036–0.087) |
| 70 (W) | 6994 | 0.051 (0.027–0.091) | 0.014 (0.007–0.024) | 0.004 (0.002–0.007) | 0.069 (0.043–0.110) |
| 80 (M) | 3490 | 0.012 (0.007–0.021) | 0.0017 (0.0010–0.0028) | 0.0001 (0.0001–0.0002) | 0.014 (0.009–0.023) |
| 80 (W) | 6538 | 0.016 (0.009–0.029) | 0.0028 (0.0015–0.0047) | 0.0003 (0.0002–0.0005) | 0.019 (0.012–0.032) |
| Whole population | 108473 | 0.37 (0.31–0.46) | 0.14 (0.11–0.17) | 0.18 (0.14–0.22) | 0.69 (0.61–0.79) |
| 40s–70s | 55798 | 0.15 (0.11–0.19) | 0.052 (0.040–0.068) | 0.044 (0.033–0.058) | 0.24 (0.20–0.29) |
