# Supplementary material for: Additional risk of diabetes exceeds the increased risk of cancer caused by radiation exposure after the Fukushima disaster
Source: PLoS One. 2017 Sep 28;12(9):e0185259. doi: 10.1371/journal.pone.0185259 (PMC5619752; doi:10.1371/journal.pone.0185259)
Supplement: S6 Table — The additional post-disaster risk was assessed. M: men; W: women. Values in parentheses represent the 2.5–97.5 percentile range. (PDF) [file pone.0185259.s007.pdf]

S6 Table.

LARs of radiation exposure-induced cancer mortality according to age. The additional post-disaster risk was assessed. M: men; W: women. Values in parentheses represent the 2.5–97.5 percentile range.

Age at the disaster	Population (persons)	LARs of radiation exposure-induced cancer mortality in each stage (10^{-3})			
		Years 1–4	Years 5–10	Years 11–	Total
0 (M)	458	0.89 (0.43–1.69)	0.34 (0.16–0.66)	0.52 (0.25–0.97)	1.76 (1.13–2.66)
0 (W)	441	1.06 (0.49–2.03)	0.41 (0.19–0.79)	0.60 (0.28–1.15)	2.06 (1.30–3.17)
5 (M)	4436	0.74 (0.35–1.41)	0.28 (0.13–0.53)	0.42 (0.21–0.78)	1.44 (0.92–2.19)
5 (W)	4261	0.87 (0.40–1.69)	0.33 (0.15–0.64)	0.48 (0.23–0.92)	1.69 (1.06–2.62)
10 (M)	5268	0.59 (0.28–1.12)	0.21 (0.10–0.40)	0.35 (0.17–0.65)	1.15 (0.74–1.76)
10 (W)	4972	0.69 (0.32–1.34)	0.25 (0.12–0.48)	0.40 (0.19–0.77)	1.35 (0.85–2.09)
20 (M)	4933	0.36 (0.18–0.68)	0.15 (0.07–0.28)	0.25 (0.12–0.45)	0.75 (0.49–1.13)
20 (W)	4470	0.42 (0.20–0.82)	0.17 (0.08–0.33)	0.28 (0.13–0.52)	0.87 (0.55–1.33)
30 (M)	6914	0.27 (0.13–0.50)	0.11 (0.05–0.20)	0.17 (0.09–0.30)	0.54 (0.36–0.80)
30 (W)	6494	0.31 (0.14–0.59)	0.12 (0.06–0.23)	0.18 (0.09–0.34)	0.61 (0.39–0.93)
40 (M)	6112	0.20 (0.10–0.36)	0.079 (0.041–0.144)	0.10 (0.05–0.18)	0.38 (0.25–0.56)
40 (W)	5927	0.22 (0.10–0.41)	0.086 (0.042–0.161)	0.11 (0.06–0.21)	0.42 (0.27–0.63)
50 (M)	7765	0.14 (0.07–0.26)	0.056 (0.029–0.099)	0.056 (0.030–0.096)	0.25 (0.17–0.38)
50 (W)	7810	0.15 (0.07–0.28)	0.058 (0.029–0.108)	0.063 (0.033–0.115)	0.27 (0.18–0.42)
60 (M)	7931	0.098 (0.053–0.174)	0.035 (0.019–0.061)	0.023 (0.013–0.040)	0.16 (0.10–0.24)
60 (W)	7867	0.10 (0.05–0.18)	0.037 (0.02–0.07)	0.029 (0.02–0.05)	0.17 (0.11–0.26)
70 (M)	5392	0.060 (0.033–0.102)	0.017 (0.010–0.029)	0.006 (0.003–0.009)	0.083 (0.053–0.126)
70 (W)	6994	0.062 (0.032–0.111)	0.020 (0.010–0.035)	0.009 (0.005–0.015)	0.090 (0.057–0.141)
80 (M)	3490	0.025 (0.014–0.043)	0.004 (0.002–0.007)	0.0005 (0.0003–0.0008)	0.030 (0.019–0.048)
80 (W)	6538	0.029 (0.015–0.051)	0.006 (0.003–0.011)	0.001 (0.001–0.002)	0.036 (0.022–0.058)
Whole population	108473	0.27 (0.22–0.33)	0.10 (0.08–0.13)	0.15 (0.12–0.18)	0.52 (0.46–0.59)
40s–70s	55798	0.13 (0.10–0.16)	0.048 (0.037–0.062)	0.049 (0.037–0.064)	0.22 (0.19–0.26)
