# Supplementary material for: Additional risk of diabetes exceeds the increased risk of cancer caused by radiation exposure after the Fukushima disaster
Source: PLoS One. 2017 Sep 28;12(9):e0185259. doi: 10.1371/journal.pone.0185259 (PMC5619752; doi:10.1371/journal.pone.0185259)
Supplement: S7 Table — (PDF) [file pone.0185259.s008.pdf]

S7 Table.

LLEs among patients with diabetes (years).

Years of incidence	Age at the disaster (men)				Age at the disaster (women)			
	40	50	60	70	40	50	60	70
Year 1	3.1	2.6	2.0	1.2	4.6	4.1	3.5	2.6
Year 2	3.0	2.5	1.9	1.2	4.5	4.0	3.4	2.5
Year 3	3.0	2.4	1.8	1.1	4.5	4.0	3.3	2.3
Year 4	2.9	2.4	1.7	1.0	4.4	3.9	3.2	2.2
Year 5	2.9	2.3	1.6	0.9	4.4	3.8	3.1	2.1
Year 6	2.8	2.2	1.5	0.8	4.3	3.8	3.0	2.0
Year 7	2.8	2.2	1.4	0.8	4.3	3.7	2.9	1.9
Year 8	2.7	2.1	1.4	0.7	4.2	3.6	2.8	1.7
Year 9	2.6	2.0	1.3	0.6	4.2	3.5	2.7	1.6
Year 10	2.6	1.9	1.2	0.6	4.1	3.5	2.6	1.5
Years 1–4	3.0	2.5	1.8	1.1	4.5	4.0	3.3	2.4
Years 5–10	2.7	2.1	1.4	0.7	4.2	3.7	2.8	1.8
