# Supplementary material for: Additional risk of diabetes exceeds the increased risk of cancer caused by radiation exposure after the Fukushima disaster
Source: PLoS One. 2017 Sep 28;12(9):e0185259. doi: 10.1371/journal.pone.0185259 (PMC5619752; doi:10.1371/journal.pone.0185259)
Supplement: S8 Table — (PDF) [file pone.0185259.s009.pdf]

S8 Table.

LARs of mortality among patients with diabetes.

Years of incidence	Age at the disaster (men)				Age at the disaster (women)			
	40	50	60	70	40	50	60	70
Year 1	0.28	0.27	0.25	0.20	0.42	0.41	0.40	0.36
Year 2	0.28	0.27	0.24	0.18	0.42	0.41	0.39	0.35
Year 3	0.28	0.26	0.24	0.17	0.41	0.41	0.39	0.34
Year 4	0.28	0.26	0.23	0.16	0.41	0.40	0.38	0.33
Year 5	0.28	0.26	0.22	0.15	0.41	0.40	0.38	0.32
Year 6	0.27	0.25	0.22	0.14	0.41	0.40	0.37	0.30
Year 7	0.27	0.25	0.21	0.13	0.41	0.40	0.37	0.29
Year 8	0.27	0.25	0.20	0.12	0.41	0.40	0.36	0.28
Year 9	0.27	0.24	0.19	0.11	0.41	0.39	0.36	0.26
Year 10	0.27	0.24	0.18	0.11	0.41	0.39	0.35	0.25
Years 1–4	0.28	0.27	0.24	0.18	0.41	0.41	0.39	0.34
Years 5–10	0.27	0.25	0.20	0.13	0.41	0.40	0.36	0.28
