# Supplementary material for: Additional risk of diabetes exceeds the increased risk of cancer caused by radiation exposure after the Fukushima disaster
Source: PLoS One. 2017 Sep 28;12(9):e0185259. doi: 10.1371/journal.pone.0185259 (PMC5619752; doi:10.1371/journal.pone.0185259)
Supplement: S9 Table — The additional post-disaster risk was assessed. Scenario 1: Emerging diabetes occurred only during the first 4 years. Scenario 2: Emerging diabetes occurred during the first 10 years. Premature incidence was considered. Scenario 3: Diabetes prevalence combined the worst cases of scenarios 1 and 2 (only emerging diabetes was considered, and no premature incidence was assumed). The values in parenthesis represent 95% confidence interval. (PDF) [file pone.0185259.s010.pdf]

S9 Table.

LLEs and LARs of mortality due to diabetes by age. The additional post-disaster risk was assessed. Scenario 1: Emerging diabetes occurred only during the first 4 years. Scenario 2: Emerging diabetes occurred during the first 10 years. Premature incidence was considered. Scenario 3: Diabetes prevalence combined the worst cases of scenarios 1 and 2 (only emerging diabetes was considered, and no premature incidence was assumed). The values in parenthesis represent 95% confidence interval.

	Population (persons)	Total LLEs (10 person-years)			LLEs (10^{-2} years)			LARs of mortality (10^{-3})		
		Years 1–4	Years 5–10	Total	Years 1–4	Years 5–10	Total	Years 1–4	Years 5–10	Total
Scenario 1										
40 (M)	6112	68 (−124–259)	0 (–)	68 (−124–259)	11.1 (−20.3–42.4)	0 (–)	11.1 (−20.3–42.4)	10.4 (−19.0–39.8)	0 (–)	10.4 (−19.0–39.8)
40 (W)	5927	−54 (−114–6)	0 (–)	−54 (−114–6)	−9.1 (−19.3–1.1)	0 (–)	−9.1 (−19.3–1.1)	−8.4 (−17.8–1.0)	0 (–)	−8.4 (−17.8–1.0)
50 (M)	7765	89 (8–170)	0 (–)	89 (8–170)	11.5 (1.1–21.9)	0 (–)	11.5 (1.1–21.9)	12.3 (1.2–23.5)	0 (–)	12.3 (1.2–23.5)
50 (W)	7810	29 (−67–125)	0 (–)	29 (−67–125)	3.7 (−8.6–16.0)	0 (–)	3.7 (−8.6–16.0)	3.8 (−8.7–16.3)	0 (–)	3.8 (−8.7–16.3)
60 (M)	7931	41 (−9–92)	0 (–)	41 (−9–92)	5.2 (−1.1–11.5)	0 (–)	5.2 (−1.1–11.5)	6.8 (−1.4–15.0)	0 (–)	6.8 (−1.4–15.0)
60 (W)	7867	39 (−5–84)	0 (–)	39 (−5–84)	5.0 (−0.7–10.7)	0 (–)	5.0 (−0.7–10.7)	5.9 (−0.8–12.5)	0 (–)	5.9 (−0.8–12.5)
70 (M)	5392	40 (25–55)	0 (–)	40 (25–55)	7.4 (4.6–10.3)	0 (–)	7.4 (4.6–10.3)	12.0 (7.4–16.6)	0 (–)	12.0 (7.4–16.6)
70 (W)	6994	26 (−7–59)	0 (–)	26 (−7–59)	3.7 (−1.0–8.4)	0 (–)	3.7 (−1.0–8.4)	5.3 (−1.4–12.0)	0 (–)	5.3 (−1.4–12.0)
Whole	108473	279 (30–528)	0 (–)	279 (30–528)	2.6 (0.3–4.9)	0 (–)	2.6 (0.3–4.9)	3.1 (0.8–5.4)	0 (–)	3.1 (0.8–5.4)
40s-70s	55798	279 (30–528)	0 (–)	279 (30–528)	5.0 (0.5–9.5)	0 (–)	5.0 (0.5–9.5)	6.1 (1.6–10.6)	0 (–)	6.1 (1.6–10.6)
Scenario 2										
40 (M)	6112	0 (−268–269)	0 (–)	0 (−268–269)	0.1 (−43.8–44.0)	0.0 (0.0–0.0)	0.1 (−43.8–44.0)	−0.6 (−43.1–41.9)	0 (–)	−0.6 (−43.1–41.9)
40 (W)	5927	−54 (−114–6)	85 (−2–173)	31 (−75–137)	−9.1 (−19.3–1.1)	14.4 (−0.4–29.1)	5.2 (−12.7–23.2)	−8.4 (−17.8–1.0)	13.8 (−0.4–28.1)	5.4 (−11.6–22.5)
50 (M)	7765	22 (−102–146)	0 (–)	22 (−102–146)	2.9 (−13.1–18.9)	0.0 (0.0–0.0)	2.9 (−13.1–18.9)	2.2 (−15.8–20.3)	0 (–)	2.2 (−15.8–20.3)
50 (W)	7810	29 (−67–125)	49 (−57–156)	78 (−65–222)	3.7 (−8.6–16.0)	6.3 (−7.3–19.9)	10.0 (−8.3–28.4)	3.8 (−8.7–16.3)	6.9 (−7.9–21.7)	10.6 (−8.7–30.0)
60 (M)	7931	41 (−9–92)	11 (−33–56)	53 (−14–120)	5.2 (−1.1–11.5)	1.4 (−4.2–7.0)	6.6 (−1.8–15.1)	6.8 (−1.4–15.0)	2.0 (−6.1–10.2)	8.8 (−2.7–20.4)
60 (W)	7867	39 (−5–84)	19 (−29–67)	59 (−7–124)	5.0 (−0.7–10.7)	2.4 (−3.7–8.5)	7.4 (−0.9–15.8)	5.9 (−0.8–12.5)	3.1 (−4.8–11.0)	9.0 (−1.3–19.3)
70 (M)	5392	40 (25–55)	0 (–)	40 (25–55)	7.4 (4.6–10.3)	0 (–)	7.4 (4.6–10.3)	12.0 (7.4–16.6)	0 (–)	12.0 (7.4–16.6)
70 (W)	6994	26 (−7–59)	0 (–)	26 (−7–59)	3.7 (−1.0–8.4)	0 (–)	3.7 (−1.0–8.4)	5.3 (−1.4–12.0)	0 (–)	5.3 (−1.4–12.0)
Whole	108473	145 (−181–471)	165 (12–317)	310 (−50–669)	1.3 (−1.7–4.3)	1.5 (0.1–2.9)	2.9 (−0.5–6.2)	1.8 (−1.2–4.9)	1.6 (0.1–3.2)	3.4 (0.0–6.9)
40s-70s	55798	145 (−181–471)	165 (12–317)	310 (−50–669)	2.6 (−3.2–8.4)	3.0 (0.2–5.7)	5.5 (−0.9–12.0)	3.5 (−2.4–9.4)	3.2 (0.1–6.2)	6.7 (0.0–13.3)

	Population (persons)	Total LLEs (10 person–years)			LLEs (10^{-2} years)			LARs of mortality (10^{-3})		
		Years 1–4	Years 5–10	Total	Years 1–4	Years 5–10	Total	Years 1–4	Years 5–10	Total
Scenario 3										
40 (M)	6112	68 (−124–259)	0 (–)	68 (−124–259)	11.1 (−20.3–42.4)	0 (–)	11.1 (−20.3–42.4)	10.4 (−19.0–39.8)	0 (–)	10.4 (−19.0–39.8)
40 (W)	5927	−54 (−114–6)	85 (−2–173)	31 (−75–137)	−9.1 (−19.3–1.1)	14.4 (−0.4–29.1)	5.2 (−12.7–23.2)	−8.4 (−17.8–1.0)	13.8 (−0.4–28.1)	5.4 (−11.6–22.5)
50 (M)	7765	89 (8–170)	0 (–)	89 (8–170)	11.5 (1.1–21.9)	0 (–)	11.5 (1.1–21.9)	12.3 (1.2–23.5)	0 (–)	12.3 (1.2–23.5)
50 (W)	7810	29 (−67–125)	49 (−57–156)	78 (−65–222)	3.7 (−8.6–16.0)	6.3 (−7.3–19.9)	10.0 (−8.3–28.4)	3.8 (−8.7–16.3)	6.9 (−7.9–21.7)	10.6 (−8.7–30.0)
60 (M)	7931	41 (−9–92)	11 (−33–56)	53 (−14–120)	5.2 (−1.1–11.5)	1.4 (−4.2–7.0)	6.6 (−1.8–15.1)	6.8 (−1.4–15.0)	2.0 (−6.1–10.2)	8.8 (−2.7–20.4)
60 (W)	7867	39 (−5–84)	19 (−29–67)	59 (−7–124)	5.0 (−0.7–10.7)	2.4 (−3.7–8.5)	7.4 (−0.9–15.8)	5.9 (−0.8–12.5)	3.1 (−4.8–11.0)	9.0 (−1.3–19.3)
70 (M)	5392	40 (25–55)	0 (–)	40 (25–55)	7.4 (4.6–10.3)	0 (–)	7.4 (4.6–10.3)	12.0 (7.4–16.6)	0 (–)	12.0 (7.4–16.6)
70 (W)	6994	26 (−7–59)	0 (–)	26 (−7–59)	3.7 (−1.0–8.4)	0 (–)	3.7 (−1.0–8.4)	5.3 (−1.4–12.0)	0 (–)	5.3 (−1.4–12.0)
Whole	108473	279 (30–528)	165 (12–317)	444 (152–736)	2.6 (0.3–4.9)	1.5 (0.1–2.9)	4.1 (1.4–6.8)	3.1 (0.8–5.4)	1.6 (0.1–3.2)	4.8 (2.0–7.5)
40s-70s	55798	279 (30–528)	165 (12–317)	444 (152–736)	5.0 (0.5–9.5)	3.0 (0.2–5.7)	8.0 (2.7–13.2)	6.1 (1.6–10.6)	3.2 (0.1–6.2)	9.3 (3.9–14.7)
