# Supplementary material for: Additional risk of diabetes exceeds the increased risk of cancer caused by radiation exposure after the Fukushima disaster
Source: PLoS One. 2017 Sep 28;12(9):e0185259. doi: 10.1371/journal.pone.0185259 (PMC5619752; doi:10.1371/journal.pone.0185259)
Supplement: S10 Table — (PDF) [file pone.0185259.s011.pdf]

S10 Table.

Details of the costs of countermeasures and their defrayers.

	Per-capita cost (JPY)	Defrayer
Restriction of food distribution		
Opportunity loss for foods	700	Central government and/or TEPCO (as compensation)
Monitoring of foods	10	Central government and/or TEPCO
Monitoring of tap water	40	TEPCO
Total	750	
Decontamination	1100000	Central government and/or TEPCO
Whole-body counter tests and interventions		
Instruments	590	Local government and central government
Consultation	5000	Local government and central government
Travel and time cost	1800	Residents
Total	7400	
Health checkup and metformin therapy for diabetes		
Health checkup	280000	Local government: 87.5%<br>Residents: 12.5%
Metformin therapy	57000	Health insurance union: ~70%<br>Residents: ~30%
Total	340000	
