# Supplementary material for: Additional risk of diabetes exceeds the increased risk of cancer caused by radiation exposure after the Fukushima disaster
Source: PLoS One. 2017 Sep 28;12(9):e0185259. doi: 10.1371/journal.pone.0185259 (PMC5619752; doi:10.1371/journal.pone.0185259)
Supplement: S11 Table — M: men; W: women. (PDF) [file pone.0185259.s012.pdf]

S11 Table.

Effects of restricted food distribution on life-years saved. M: men; W: women.

Age at the disaster	Population (persons)	Total life-years saved (person-years)	Life-years saved (10^{-5} years)
0 (M)	1218	0.006	0.53
0 (W)	1143	0.008	0.72
5 (M)	11616	0.21	1.8
5 (W)	10951	0.25	2.3
10 (M)	14959	0.37	2.4
10 (W)	14355	0.49	3.4
20 (M)	14095	0.33	2.4
20 (W)	14517	0.44	3.0
30 (M)	18998	0.32	1.7
30 (W)	19350	0.41	2.1
40 (M)	17972	0.22	1.2
40 (W)	18578	0.26	1.4
50 (M)	19075	0.16	0.82
50 (W)	19762	0.18	0.89
60 (M)	19561	0.095	0.48
60 (W)	21036	0.11	0.50
70 (M)	13541	0.031	0.23
70 (W)	16764	0.041	0.24
80 (M)	9418	0.007	0.07
80 (W)	15083	0.013	0.08
Whole population	291992	3.94	1.3
