# Supplementary material for: Additional risk of diabetes exceeds the increased risk of cancer caused by radiation exposure after the Fukushima disaster
Source: PLoS One. 2017 Sep 28;12(9):e0185259. doi: 10.1371/journal.pone.0185259 (PMC5619752; doi:10.1371/journal.pone.0185259)
Supplement: S12 Table — (PDF) [file pone.0185259.s013.pdf]

S12 Table.

Unit and per-capita costs for restricted food distribution.

	Unit cost (JPY/kg)	Per-capita cost (JPY)
Rice	230[a]	210
Dairy products	54[b]	19
Milk	54[b]	120
Tea	140[b]	1.4
Turnips	72[c]	6.8
Spinach	260[a]	65
Garland chrysanthemum and ging-geng-cai	310[a]	29
Mustard spinach and non-heading lettuce	130[c]	47
Heading leafy vegetables	31[c]	21
Broccoli and cauliflower	170[a]	50
Kiwifruit	180[c]	0.48
Chestnut	120[c]	0.04
Bamboo shoots	47[c]	0.36
Mushrooms	580[a]	20
Beef	470[d]	89
Wild *ayu*, wild Japanese dace and wild landlocked *masu* salmon	1410[c]	14
Total		700

a: [22]; b: [23]; c: [24]; d: [25].
