# Supplementary material for: Additional risk of diabetes exceeds the increased risk of cancer caused by radiation exposure after the Fukushima disaster
Source: PLoS One. 2017 Sep 28;12(9):e0185259. doi: 10.1371/journal.pone.0185259 (PMC5619752; doi:10.1371/journal.pone.0185259)
Supplement: S13 Table — (PDF) [file pone.0185259.s014.pdf]

S13 Table.

Effects of decontamination on life-years saved.

	Population (persons)	Total life-years saved (person-years)	Life-years saved (10^{-2} years)
0 (M)	1218	5.1	1.7
0 (W)	1143	6.8	2.4
5 (M)	11616	40	1.4
5 (W)	10951	52	1.9
10 (M)	14959	36	1.1
10 (W)	14355	46	1.5
20 (M)	14095	21	0.69
20 (W)	14517	26	0.94
30 (M)	18998	21	0.47
30 (W)	19350	26	0.61
40 (M)	17972	12	0.30
40 (W)	18578	14	0.37
50 (M)	19075	8.9	0.17
50 (W)	19762	11	0.21
60 (M)	19561	4.4	0.084
60 (W)	21036	5.3	0.10
70 (M)	13541	1.1	0.030
70 (W)	16764	1.8	0.039
80 (M)	9418	0.1	0.006
80 (W)	15083	0.4	0.009
Whole population	291992	340	0.48
