# Supplementary material for: Additional risk of diabetes exceeds the increased risk of cancer caused by radiation exposure after the Fukushima disaster
Source: PLoS One. 2017 Sep 28;12(9):e0185259. doi: 10.1371/journal.pone.0185259 (PMC5619752; doi:10.1371/journal.pone.0185259)
Supplement: S14 Table — M: men; W: women. (PDF) [file pone.0185259.s015.pdf]

S14 Table.

Effects of whole-body counter tests and interventions on life-years saved. M: men; W: women.

Age	Effective dose in first whole-body counter screening (mSv/year)	LYS (10^{-4} years)
70 (M)	0.435	1.8
66 (W)	0.263	2.2
71 (M)	0.682	2.5
64 (M)	0.971	7.9
74 (M)	0.759	1.8
74 (W)	0.301	0.99
73 (M)	0.209	0.57
69 (W)	0.141	0.82
