# Supplementary figures and images for: Additional risk of diabetes exceeds the increased risk of cancer caused by radiation exposure after the Fukushima disaster
Source: PLoS One. 2017 Sep 28;12(9):e0185259. doi: 10.1371/journal.pone.0185259 (PMC5619752; doi:10.1371/journal.pone.0185259)

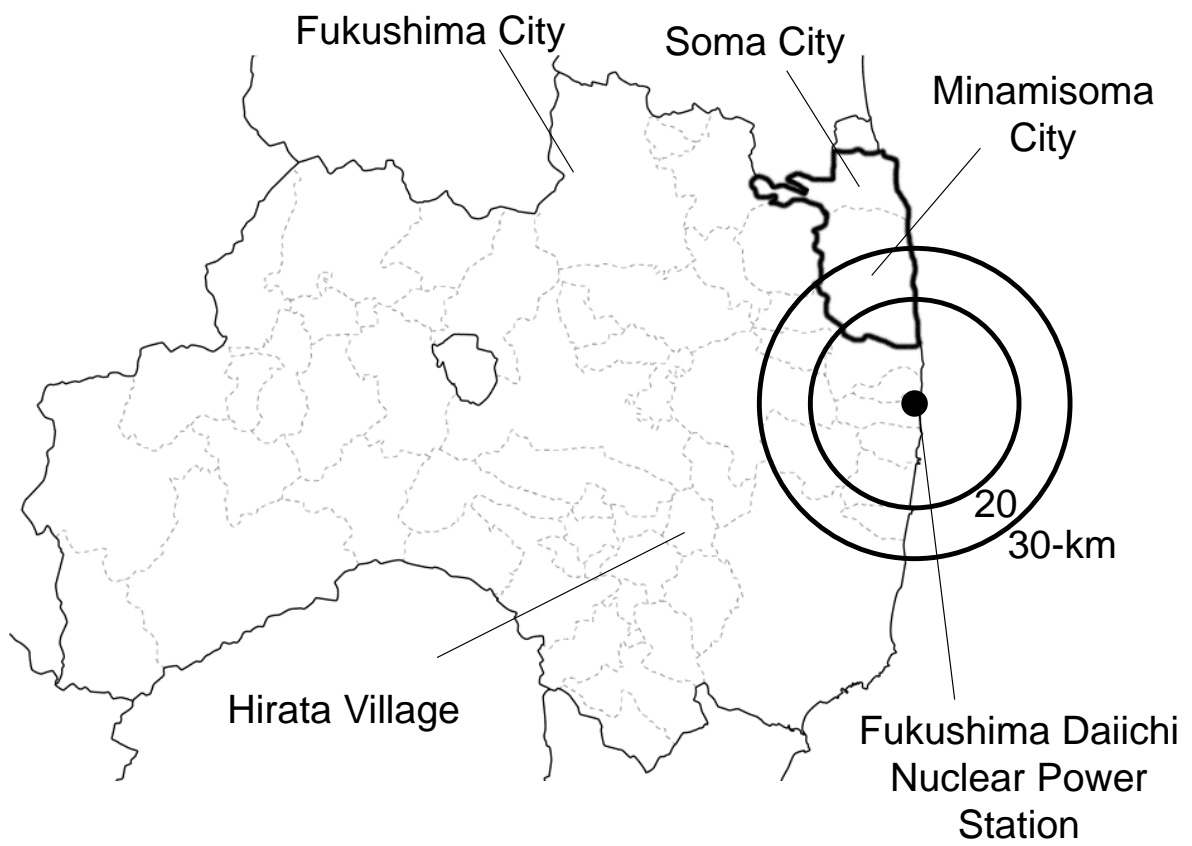

**S1 Figure.**

Locations of Minamisoma City and Soma City.

Supplement: S1 Fig — (PDF) [file pone.0185259.s016.pdf]
