# Supplementary material for: Additional risk of diabetes exceeds the increased risk of cancer caused by radiation exposure after the Fukushima disaster
Source: PLoS One. 2017 Sep 28;12(9):e0185259. doi: 10.1371/journal.pone.0185259 (PMC5619752; doi:10.1371/journal.pone.0185259)
Supplement: S2 Fig — 0 y and ≥20 y indicate ages at the time of the disaster. M: men. (PDF) [file pone.0185259.s017.pdf]

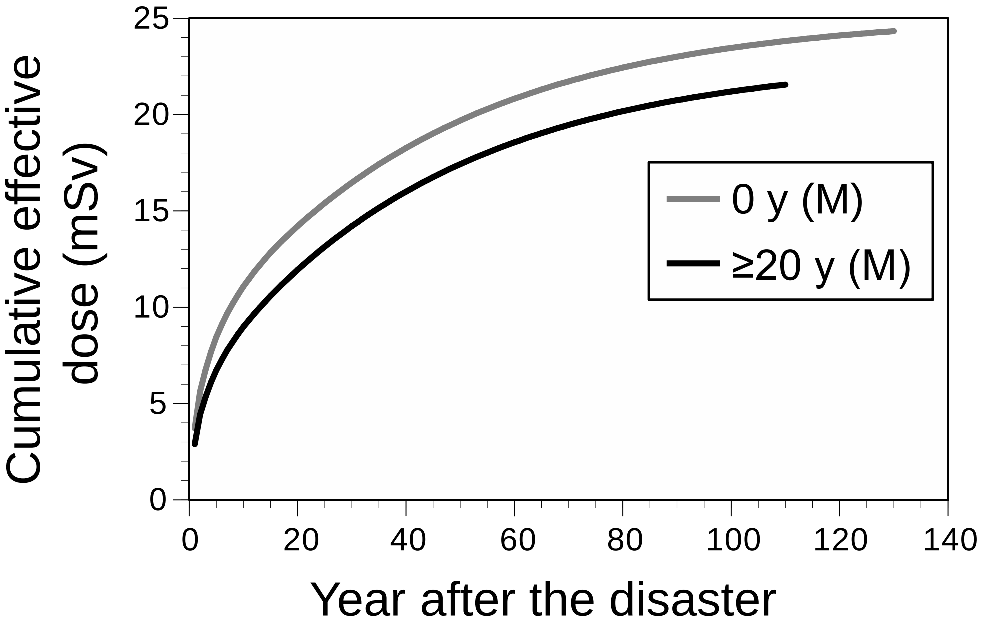

S2 Figure.

Cumulative changes in radiation exposure. 0 y and ≥20 y indicate ages at the time of the disaster. M: men.
