# Supplementary material for: Additional risk of diabetes exceeds the increased risk of cancer caused by radiation exposure after the Fukushima disaster
Source: PLoS One. 2017 Sep 28;12(9):e0185259. doi: 10.1371/journal.pone.0185259 (PMC5619752; doi:10.1371/journal.pone.0185259)
Supplement: S3 Fig — (A) Temporal changes in radiocesium levels in the body and (B) the correlation between measured and predicted radiocesium levels at the second and third measurements. Individual plots represent residents with high levels of internal contamination after whole-body counter screening. The lines in A represent reduction according to the biological half-life, assuming that radiocesium intake stopped 30 d after whole-body counter screening. Under the same assumption, the predicted radiocesium values in B were determined according to the biological half-life. (PDF) [file pone.0185259.s018.pdf]

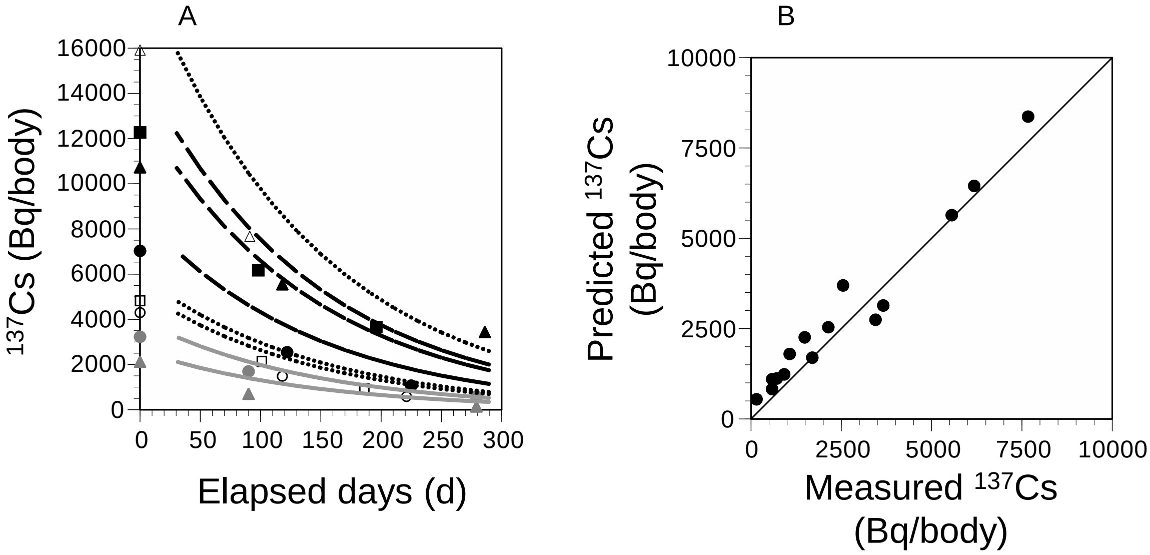

S3 Figure.

(A) Temporal changes in radiocesium levels in the body and (B) the correlation between measured and predicted radiocesium levels at the second and third measurements. Individual plots represent residents with high levels of internal contamination after whole-body counter screening. The lines in *A* represent reduction according to the biological half-life, assuming that radiocesium intake stopped 30 d after whole-body counter screening. Under the same assumption, the predicted radiocesium values in *B* were determined according to the biological half-life.
